# Discovery of high-confidence human protein-coding genes and exons by whole-genome PhyloCSF helps elucidate 118 GWAS loci

**DOI:** 10.1101/gr.246462.118

**Published:** 2019-12

**Authors:** Jonathan M. Mudge, Irwin Jungreis, Toby Hunt, Jose Manuel Gonzalez, James C. Wright, Mike Kay, Claire Davidson, Stephen Fitzgerald, Ruth Seal, Susan Tweedie, Liang He, Robert M. Waterhouse, Yue Li, Elspeth Bruford, Jyoti S. Choudhary, Adam Frankish, Manolis Kellis

**Affiliations:** 1 European Molecular Biology Laboratory, European Bioinformatics Institute, Wellcome Genome Campus, Hinxton, Cambridge CB10 1SD, United Kingdom;; 2 MIT Computer Science and Artificial Intelligence Laboratory, Cambridge, Massachusetts 02139, USA;; 3 Broad Institute of MIT and Harvard, Cambridge, Massachusetts 02142, USA;; 4 Functional Proteomics, Division of Cancer Biology, Institute of Cancer Research, London SW7 3RP, United Kingdom;; 5 Wellcome Trust Sanger Institute, Hinxton, Cambridge CB10 1SA, United Kingdom;; 6 Department of Haematology, University of Cambridge, Cambridge CB2 0PT, United Kingdom;; 7 Department of Ecology and Evolution, University of Lausanne, Lausanne 1015, Switzerland;; 8 Swiss Institute of Bioinformatics, Lausanne 1015, Switzerland

## Abstract

The most widely appreciated role of DNA is to encode protein, yet the exact portion of the human genome that is translated remains to be ascertained. We previously developed PhyloCSF, a widely used tool to identify evolutionary signatures of protein-coding regions using multispecies genome alignments. Here, we present the first whole-genome PhyloCSF prediction tracks for human, mouse, chicken, fly, worm, and mosquito. We develop a workflow that uses machine learning to predict novel conserved protein-coding regions and efficiently guide their manual curation. We analyze more than 1000 high-scoring human PhyloCSF regions and confidently add 144 conserved protein-coding genes to the GENCODE gene set, as well as additional coding regions within 236 previously annotated protein-coding genes, and 169 pseudogenes, most of them disabled after primates diverged. The majority of these represent new discoveries, including 70 previously undetected protein-coding genes. The novel coding genes are additionally supported by single-nucleotide variant evidence indicative of continued purifying selection in the human lineage, coding-exon splicing evidence from new GENCODE transcripts using next-generation transcriptomic data sets, and mass spectrometry evidence of translation for several new genes. Our discoveries required simultaneous comparative annotation of other vertebrate genomes, which we show is essential to remove spurious ORFs and to distinguish coding from pseudogene regions. Our new coding regions help elucidate disease-associated regions by revealing that 118 GWAS variants previously thought to be noncoding are in fact protein altering. Altogether, our PhyloCSF data sets and algorithms will help researchers seeking to interpret these genomes, while our new annotations present exciting loci for further experimental characterization.

It has been almost two decades since the first high-quality sequences from the human genome became available (International Human Genome Sequencing Consortium 2001; Venter et al. 2001). Nonetheless, efforts to decipher the information contained in our genome remain ongoing, and a key challenge is to identify regions that encode protein-coding sequences (CDSs). At present, the two main human gene annotation projects, Ensembl/GENCODE (Harrow et al. 2012; Frankish et al. 2018; Zerbino et al. 2018) (henceforth GENCODE) and RefSeq (O'Leary et al. 2016), as well as the UniProt protein resource (The UniProt Consortium 2019), disagree on the number of human protein-coding genes (Abascal et al. 2018), and even when a gene is agreed to be protein coding, it is often unclear which transcripts within the locus are translated (Mudge et al. 2013; Tress et al. 2017). It has been historically challenging to obtain protein sequences in the laboratory in a high-throughput manner, and it remains far easier to describe the structure of transcribed regions than to ascertain their coding potential. The number of experimentally derived peptide sequences found in online repositories such as PRIDE (Vizcaíno et al. 2016) has increased substantially in recent years, and although such data sets have been used to discover novel proteins in genomes including human (Slavoff et al. 2012), difficulties remain in the creation and interpretation of high-quality “proteogenomic” data sets (Nesvizhskii 2014). Meanwhile, ribosome profiling (RP) circumvents the experimental challenges in working with proteins, capturing sequence from the region of an RNA molecule that is attached to a ribosome (Ingolia et al. 2009). These data have been used to suggest the biological relevance of thousands of currently unannotated vertebrate open reading frames (ORFs) (Ingolia et al. 2011; Bazzini et al. 2014; Fields et al. 2015; Mackowiak et al. 2015; Raj et al. 2016). Nonetheless, it remains unclear to what extent ribosome attachment shows production of a functional protein, that is, one that makes a direct contribution to physiology (Bazzini et al. 2014), because ORFs can also undergo translation as part of gene regulation mechanisms, and a proportion of attachments could be stochastic “noise” (Guttman et al. 2013; Jackson and Standart 2015; Johnstone et al. 2016; Raj et al. 2016).

CDSs can also be identified through sequence conservation, and both the ratio of nonsynonymous to synonymous substitutions (*d*_N_/*d*_S_) and codon substitution frequencies can be diagnostic of protein evolution (Lin et al. 2008). The power of such “comparative annotation” has increased in recent years as the number of vertebrate genome sequences available has moved from single to triple figures. Previously, we developed Phylogenetic Codon Substitution Frequencies (PhyloCSF) to support CDS annotation based on multispecies genome alignments (Lin et al. 2011). PhyloCSF determines whether a given alignment is likely to represent a functional, conserved protein-coding sequence by determining its likelihood ratio under coding and noncoding models of evolution. Unlike the traditional *d*_N_/*d*_S_ test, PhyloCSF uses precomputed substitution frequencies for every possible pair of codons, trained on whole-genome data. A particular advantage of PhyloCSF is that it can classify short portions of a CDS in isolation from the full sequence, which is necessary when considering individual exons.

We previously showed the ability of PhyloCSF and its predecessor, CSF, to add CDS annotation to genomes within the *Schizosaccharomyces* (Lin et al. 2011) and *Drosophila* lineages (Lin et al. 2007; The modENCODE Consortium et al. 2010; Jungreis et al. 2011, 2016), and also to identify novel human and mouse protein-coding genes based on the alignment of 29 mammalian genomes (Lindblad-Toh et al. 2011). Meanwhile, Mackowiak et al. (2015) used PhyloCSF to find 2000 candidates for conserved short ORFs (sORFs) in the human, mouse, zebrafish, *Drosophila melanogaster*, and *Caenorhabditis elegans* genomes, while Bazzini et al. (2014) used PhyloCSF to score ORFs within a set of RP translations observed in human and zebrafish. However, the efficacy of PhyloCSF has thus far been judged on its ability to recover *known* CDSs, and few of the novel CDSs predicted by these publications have undergone rigorous validation. GENCODE seeks to describe the true set of human protein-coding genes, not a larger set of plausible models. The inclusion of false CDSs could have undesirable consequences for GENCODE's users, for example, in the interpretation of clinical variants. Thus, externally published novel CDSs are always manually reassessed according to GENCODE criteria. Although we have found that such publications may report an excess of false-positive novel protein-coding genes (Uszczynska-Ratajczak et al. 2018), they are also likely to have underreported the set of true-positives awaiting discovery because they generally targeted existing transcript catalogs, reducing the discovery space to a few percent of the genome sequence. This is also generally true of mass spectrometry and RP-based projects.

Our goals in the current study were to develop algorithms that would allow PhyloCSF to be applied across whole genomes to find and prioritize candidate novel protein-coding regions, even in regions previously thought to be intergenic; to develop a workflow to enable manual annotators to investigate those candidates using modern transcriptomic and mass spectrometry data sets, as well as cross-species comparative annotation; and to use the resulting improved annotations to recharacterize “noncoding” variants associated with traits or diseases as protein altering.

We use the term “novel” or “new discovery” to describe a coding gene, coding sequence, or pseudogene that, at the time it was made publicly available by GENCODE, was not considered to be coding or, respectively, pseudogenic in the species under consideration in any of the major gene catalogs, or, as far as we could determine, in the peer-reviewed literature. By “novel” we do *not* mean de novo, that is, arising from noncoding sequence (Schlötterer 2015); in fact, many of these sequences have known orthologs in other species or paralogs in the species under consideration.

## Results

### Whole-genome PhyloCSF finds candidate novel coding regions

To find novel coding genes, coding exons, and pseudogenes, we created a ranked list of candidate genomic regions that have the evolutionary signature of coding regions but were not previously annotated as coding or pseudogenes. Because transcriptional evidence for such regions might be incomplete or missing, we used a whole-genome method unbiased by known transcription.

We first calculated the PhyloCSF score of every codon of the hg38/GRCh38 human genome reference assembly in each of the six reading frames using alignments of 29 mammalian genomes. Each codon gets a positive score if the alignment of that codon is more likely to have arisen under a model of protein-coding evolution than under a model of noncoding evolution. Because individual codon alignments do not have enough information to distinguish coding from noncoding evolution with any confidence, we combined scores of nearby codons using a hidden Markov model (HMM) with states representing coding and noncoding regions. The intervals in which the most likely path through the HMM is in the coding state define a set of 596,426 genomic regions, “PhyloCSF Regions,” that likely include almost all conserved coding regions, both known and novel, that generate a PhyloCSF signal, as well as many false positives.

**Figure 1.**
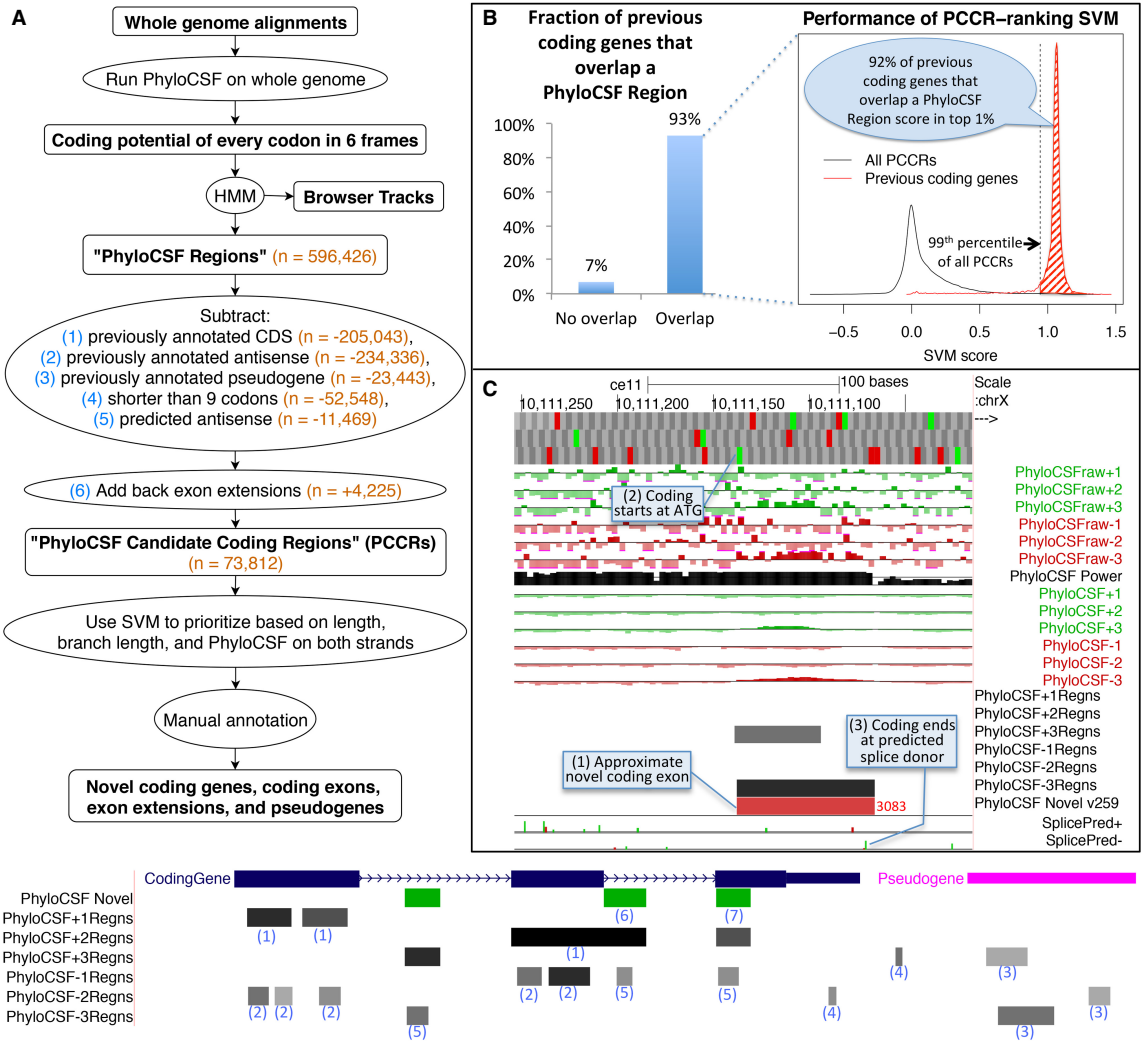
Computing PhyloCSF Candidate Coding Regions (PCCRs). (*A*) Flow chart of overall process. Numbers in orange are counts for the human hg38 assembly relative to the GENCODE v23 gene set. The hypothetical browser image at the *bottom* illustrates how the PhyloCSF Regions list is pruned to define PCCRs. In the vicinity of a coding gene (blue) and a pseudogene (pink), we initially have a set of intervals in each of the six possible reading frames (“PhyloCSF Regions”) that are more likely to be in the coding state than noncoding state of the HMM (gray-scale intervals in the six “PhyloCSF*Regns” tracks). We then exclude any that overlap known coding genes in the same frame (1) or anti-sense frame (2) or that overlap known pseudogenes in any frame on either strand (3). Next, we exclude regions less than nine codons long (4) and regions predicted by our antisense SVM to be likely antisense regions (5). Finally, we add back nonoverlapping fragments of PhyloCSF Regions that partly overlap annotations because these could be extensions of known exons (6). The resulting PCCRs are shown in green. These sometimes overlap known coding regions, and this is an indication that the PhyloCSF signal is in a different frame from the annotated one (7). The resulting PCCRs are then ranked by an SVM and investigated by expert manual annotators to find novel coding regions and pseudogenes. (*B*) Performance on previously annotated coding genes. Column chart on the *left* shows the fraction (93%) of GENCODE v23 coding genes that overlap at least one PhyloCSF Region; the remaining 7% could not have been identified by our workflow. Density plot on the *right* measures the efficiency of our PCCR-ranking SVM by showing SVM scores for all PCCRs (black) and scores of the highest-scoring PhyloCSF Region that overlaps each GENCODE v23 coding gene that overlaps at least one PhyloCSF Region (red). For 92% of such coding genes, the score is in the 99th percentile of scores of PCCRs (shaded area), indicating that manual examination of the top-ranked 1% of PCCRs would have uncovered each of these coding genes if it had not been known previously, and suggesting that most true *novel* coding genes could be identified by examining the best ranking PCCRs. (*C*) PhyloCSF tracks in UCSC Genome Browser showing the “−” strand of *C*. *elegans* Chromosome X. *Upper* six green and red “PhyloCSFraw” tracks show the raw PhyloCSF score for each codon in each of six reading frames. The black “PhyloCSF power” track indicates the relative branch length of the local alignment, a measure of the statistical power available to PhyloCSF; there is near full alignment for the first approximately three-fourths of the track, but then there are fewer aligned species for the remaining one-fourth. Codons having relative branch length less than 0.1 show no scores. The next six green and red “PhyloCSF” tracks show the PhyloCSF scores smoothed by the HMM. The six “PhyloCSF*Regns” tracks show PhyloCSF Regions, with gray scale indicating the maximum probability of coding. The “PhyloCSF novel” track shows the PCCRs in all six frames combined into a single track with green and red intervals indicating the plus and minus strands, respectively, and with the rank of the region within the list of PCCRs shown next to the region, with lower ranks indicating stronger likelihood of coding. The two “Splice Pred” tracks show splice donor (green) and acceptor (red) predictions at GT and AG dinucleotides, respectively, on the plus and minus strands, with the height of each bar indicating the strength of the splice prediction. In the example shown, the tracks allow us to conjecture that there is a novel coding exon on the minus strand roughly coinciding with the 3083rd PCCR (1), extending from the ATG indicated by the small green rectangle in the third base position track at the top (2) up to the green splice donor prediction in the “SplicePred−” track (3).

To restrict our list to *novel* regions, we excluded 205,043 PhyloCSF Regions overlapping protein-coding sequences in the same frame that were annotated in GENCODE v23. We also excluded 234,336 regions overlapping annotated coding sequences in the “antisense frame” (the frame on the opposite strand that shares the third codon position) and 23,443 overlapping pseudogenes, because PhyloCSF often reports a protein-coding signal on their alignments even though the locus is no longer protein coding. We excluded 52,548 regions shorter than nine codons because the signal on such short regions is unreliable. To eliminate regions that are antisense to *novel* coding regions, we trained a support vector machine (SVM) to distinguish PhyloCSF Regions translated on their strand from those translated on the opposite strand, using the PhyloCSF scores on the two strands and the region length (see Methods; Supplemental Fig. S1). We excluded 11,469 regions that our SVM found to be considerably more likely to be coding on the other strand. Finally, for regions that were excluded because they partially overlap an annotation, we added back the portion that does not overlap, provided it is at least nine codons long and satisfies our antisense condition. There were 4225 such fragments, which could be 5′ extensions of annotated ORFs or extensions of known exons. This left us with 73,812 “PhyloCSF Candidate Coding Regions,” henceforth “PCCRs” (Fig. 1A).

Seeking novel coding sequence in a whole-genome scan is a needle-in-a-haystack problem. Known coding sequences comprise <0.25% of the six-frame translation of the human genome, and *novel* coding sequences presumably comprise much less. Consequently, despite the high specificity of PhyloCSF, we expect most of our PCCRs to be false positives. To determine which PCCRs are most likely to be true novel protein-coding regions, we ranked them using another SVM, this one trained to distinguish true coding PhyloCSF Regions from false positives using PhyloCSF scores on the two strands, the length of the region, and the branch length of the phylogenetic tree of species present in its local alignment (see Methods; Supplemental Fig. S1). Our algorithm considers PCCRs having lower ranks to be more likely to be real coding regions.

To evaluate our ranking, we calculated the distribution of SVM scores of previously annotated coding genes (Fig. 1B) and corresponding ranks, where the “rank of a novel coding gene” is defined to be the lowest rank of any PCCR that overlaps its CDS in the same frame, and the “rank of an annotated coding gene” is the rank it would have had if it had not been previously annotated, that is, if we had not excluded PhyloCSF Regions overlapping that particular gene when constructing the PCCRs. We found that 93% of coding genes annotated in GENCODE v23 would have overlapped a PCCR, and 92% of *those* would have ranks among the best-ranked 1% of PCCRs, suggesting that most true *novel* coding genes could be discovered by examining the best-ranked PCCRs, although PCCRs might not cover the entire CDS so further work would be needed to fully define the transcript models and CDS. Many higher-ranked regions could also indicate novel coding exons, extensions, and pseudogenes.

To facilitate the use of our whole-genome PhyloCSF scan to distinguish protein-coding regions, we created a track hub for the UCSC and Ensembl Genome Browsers (Casper et al. 2017; Zerbino et al. 2018) with tracks for the raw PhyloCSF score of every codon, the HMM-smoothed scores, the PhyloCSF Regions, the PCCRs, and splice site predictions using the maximum entropy method (Fig. 1C; Yeo and Burge 2004). We have also created browser tracks and PCCR lists for mouse, chicken, fly (*D*. *melanogaster*), worm (*C*. *elegans*), and mosquito (*Anopheles gambiae*). The details page for each PCCR includes a link to view the color-coded alignment of the region in CodAlignView (https://data.broadinstitute.org/compbio1/cav.php), and other relevant information. The PhyloCSF tracks differ from other conservation browser tracks such as phyloP (Pollard et al. 2010) and phastCons (Siepel et al. 2005) in that the PhyloCSF tracks represent a signal of constraint specifically for protein-coding function, whereas the signal represented by other tracks is independent of the cellular function imposing the constraint (Supplemental Fig. S2).

### Manual annotation of PhyloCSF regions

To find and annotate novel protein-coding regions, we manually examined many of the best-scoring PCCRs, clustered by chromosomal position. First, we focused on the 658 clusters that contained all of the top 1000 ranked PCCRs. Second, we targeted the complete set of long intergenic noncoding RNA (lincRNA) models that overlapped PCCRs of any rank, in order to find misannotated noncoding genes. Third, in order to investigate PCCRs in intergenic space, we analyzed all remaining clusters up to rank 2200 that did not overlap any existing GENCODE annotation. Finally, we investigated several ad hoc clusters not tagged by PCCRs in the above three categories during preliminary efforts to compare the consequences of using different alignments (see Supplemental Methods section “PhyloCSF and browser tracks”).

Annotation was performed in accordance with the HAVANA guidelines for the GENCODE project (see Methods). However, an expanded approach was developed for this work that included a broad range of short-read and long-read data sets, plus detailed “comparative annotation,” including equivalent manual annotation of the mouse genome where possible and manual analysis of coding potential in additional vertebrate genomes (see Supplemental Methods section “Manual annotation overview”).

### 144 protein-coding genes and 228 kb of CDS added to GENCODE

Guided by these clusters of PCCRs, we added 144 new protein-coding genes to human GENCODE (Supplemental Data S2) and additional CDSs within 236 previously annotated protein-coding genes, adding a total of 228,271 bp of CDSs. We also added 169 new pseudogenes to GENCODE and made extensions to 35 existing pseudogenes. We released each annotation to the public via the GENCODE Annotation Updates trackhub as soon as it was added, and included it in the next GENCODE release, beginning with version 24 in 2015. In what follows, when we discuss what was previously known about an annotation, we mean prior to its first public release by GENCODE. The PCCR clusters analyzed and the resulting annotations are reported in Supplemental Data S1, and detailed information about each of the PCCRs in these clusters is reported in Supplemental Data S6. Supplemental Table S1 shows counts of PCCRs among the top-ranked 1000 that resulted in each kind of annotation, broken down by transcript region (overlapping CDS, extension of CDS, UTRs, etc.).

The 144 genes were classified as protein coding because we believe that is the most likely interpretation of their functionality at the present time. In each case, we were able to support the protein-coding status by producing either a multispecies or multiparalog protein-sequence alignment, but we recognize that the true test of functionality for these loci will take place in the laboratory (Mudge et al. 2013). We note that PhyloCSF does not determine the transcript model containing the complete ORF and may not even demarcate the entire translation; even a deeply conserved protein-coding gene may not have all codons or exons marked by PhyloCSF signals (see *EDDM13*) (Supplemental Fig. S3A). Furthermore, a PhyloCSF signal indicates that a region has evolved at some point in the past as protein-coding sequence and does not rule out that it has been pseudogenized. This is important because vertebrate genomes are replete with pseudogenes (see below) (Pei et al. 2012; Sisu et al. 2014).

### Properties of the newly added protein-coding genes

**Figure 2.**
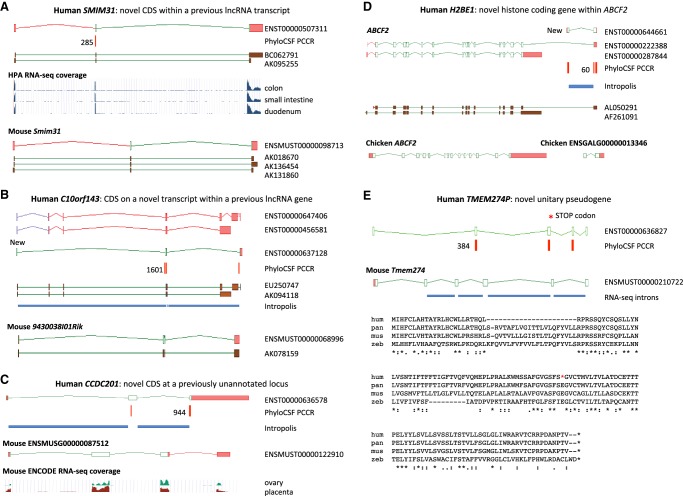
Novel protein-coding loci. Browser images show CDSs (open green rectangles), UTRs (pink), supporting PCCRs (red), top rank (black), cDNA evidence (brown), and RNA-seq–supported introns (blue rectangles). Additional transcript models omitted for clarity. Multispecies protein alignments showing conservation of complete ORFs are in Supplemental Figure S4. (*A*) Novel coding gene *SMIM31*, previously a cDNA-supported GENCODE lincRNA, was changed to protein coding without a change of transcript structure owing to a 71-aa CDS (ENST00000507311) conserved to coelacanth. The protein-coding cDNA-supported ortholog was added to mouse GENCODE (*Smim31*). PhyloCSF does not detect coding potential in the second coding exon, but multispecies protein alignment and preponderance of 3-mer indels provide evidence this exon is coding. Human Protein Atlas (HPA) RNA-seq and human and mouse FANTOM5 CAGE data show high transcription in gastrointestinal tissues. (*B*) Novel coding gene *C10orf143* was previously a GENCODE lncRNA (*LINC00959*), with two cDNA-derived models (ENST00000647406 and ENST00000456581). Discovery of the 108-aa CDS required adding a transcript model (ENST00000637128), supported by Intropolis short-read data. The original lncRNA transcripts have been reannotated as nonsense-mediated decay targets (purple ORFs), based on a premature stop codon in a cassette exon. The orthologous cDNA-supported mouse locus had previously been recognized as protein coding (*9430038I01Rik*). The gene has a broad expression profile in both species. (*C*) *CCDC201* is a novel human gene with a 187-aa CDS conserved to birds, previously missed owing to lack of spliced cDNA or EST evidence. The ancestral stop codon has been lost in rodents, adding a 30-aa extension in novel mouse protein-coding gene ENSMUSG00000087512. Introns are supported by Intropolis short-read RNA-seq, limited to female reproductive tissues and certain developmental cells. Mouse ENCODE RNA-seq supports placenta and ovary expression only, and the mouse locus (in the guise of a ncRNA) had previously been identified as a target for the germ cell–specific transcription factor *Figla* (Joshi et al. 2007). (*D*) *H2BE1* is a novel histone HB2 family member protein-coding gene with a 122-aa CDS (model ENST00000644661), whose first exon was identified in this study. Intropolis supports the transcript structure, with expression limited to oocytes and embryonic cells (e.g., SRR499827). Human FANTOM5 CAGE data lacks experiments from developmental stages, which may explain the absence of TSS evidence. Overlapping model ENST00000222388 had previously been annotated as an alternative transcript of *ABCF2* (ancestral CDS represented by model ENST00000287844) based on cDNA AL050291, with putative translation in the shared exon following the coding frame of *ABCF2*. PhyloCSF indicates that the 122-aa CDS is translated in a different frame, so the translation of ENST00000222388 is potentially spurious. Although the 122-aa CDS is conserved to birds, the locus has apparently been lost in rodents. There is no evidence for transcriptional connectivity between the orthologous Ensembl chicken models *ABCF2* and ENSGALG00000013346 (*bottom*). ENST00000222388 has been reclassified as a “readthrough” transcript, and Intropolis data indicate that such readthrough between human *ABCF2* and *H2BE1* is rare. (*E*) *TMEM274P* is a novel human unitary pseudogene, orthologous to novel mouse protein-coding gene *Tmem274*. CDS alignments to RefSeq models such as scallop LOC110448246 and trichoplax XP_002113670.1 suggest this gene may predate vertebrate evolution, although orthology is presumptive owing to lack of synteny beyond coelacanth. The gene has at best weak expression data in all species examined, but all but one of the mouse splice junctions is supported by minimal ENCODE RNA-seq data from pooled sources, and all splice sites display mammalian conservation. An alignment of human (hum) to chimp (pan), with outgroups mouse (mus) and zebrafish (zeb), shows that human has a premature stop codon that is not a known SNP in the fourth exon of the ancestral CDS (red asterisk in diagram and alignment) and has also lost the second coding exon (large gap in human sequence); both events are unique to human. The zebrafish sequence in the alignment is from XP_017212190, and the chimp translation is from the genome sequence.

Several properties of the 144 newly added protein-coding genes help explain why they were not found previously. First, the genes are enriched for small CDSs: 50 translations are under 100 aa, and the median size is only 140.5 aa; less than half of the 387-aa median of all GENCODE CDSs. Two examples are *SMIM31* (Fig. 2A) and *SMIM41* (Supplemental Fig. S3B); both CDSs were discovered within existing “noncoding” transcript models. Small CDSs are harder to identify in both manual and computational annotation pipelines (Mudge and Harrow 2016), and this problem is confounded by the fact that 28 of these 50 loci are single-exon genes. It is also probable that protein size thwarted our proteogenomics pipeline (see below), as small proteins may be harder to identify in “shotgun” mass spectrometry experiments (Nesvizhskii 2014).

Second, 78 out of 144 protein-coding loci were missed owing to a previous lack of transcript evidence. Although most GENCODE annotation is based on cDNA and EST libraries, our new workflow integrated multiple modern transcriptomic data sets. In 20 instances, the CDS was discovered after an existing “noncoding” GENCODE model was extended to incorporate the entire reading frame. In 15 other cases, CDS annotation required the discovery of an alternatively spliced transcript within a “noncoding” locus, as illustrated by *C10orf143* (Fig. 2B) and *EDDM13* (Supplemental Fig. S3A). In 44 cases, the protein-coding gene was entirely new to GENCODE—that is, it was not previously found as a lncRNA or pseudogene—with the prior absence of most of these genes being because of their restricted expression (Supplemental Data S2), as illustrated by *C1orf232* (Supplemental Fig. S3C) and *CCDC201* (Fig. 2C). Transcription of *C1orf232* appears to be limited to brain and eye tissues in human and mouse, and *CCDC201* is apparently transcribed only in female reproductive tissues and early developmental cells.

Finally, 13 protein-coding genes were identified within the UTRs of extant protein-coding loci. *H2BE1* is a particularly exciting discovery, being a novel histone protein with expression apparently limited to early development (Fig. 2D). In nine 5′ UTR cases, transcriptomic data indicate that the new CDS and the previously known downstream CDS consistently share the same RNA molecule, as illustrated by the CDS identified within the *ALDOA* 5′ UTR (Supplemental Fig. S3D). UTR-associated ORFs are extensively detected in vertebrate RP studies (Ingolia et al. 2009, 2011). However, it remains unclear what proportion of these are regulatory ORFs that do not produce functional peptides and instead compete with the canonical CDS for ribosome binding, or else are simply stochastic interactions (Calvo et al. 2009; Bazzini et al. 2014; Johnstone et al. 2016). In contrast, PhyloCSF detects the evolutionary signature of function at the amino acid level, so UTR ORFs identified by PhyloCSF are highly likely to be CDSs that produce functional peptides. In fact, our 5′ UTR–associated examples include three cases in which protein existence has been confirmed by others through laboratory work: within the 5′ UTRs of *MIEF1* (Rathore et al. 2018), *MKKS* (Akimoto et al. 2013), and *RAB34* (Zougman et al. 2011). The CDS within the *MKKS* 5′ UTR produces a mitochondrial protein, whereas *MKKS* itself is involved in cytokinesis. This observation is a reason why GENCODE chose to represent the UTR-associated CDS as distinct protein-coding genes.

### Novel protein-coding genes do not always get high SVM scores

Among the clusters containing the 1000 highest-scoring PCCRs, 81.6% led to some annotation update, whereas this was true of only 38.1% of the less well ranked clusters we investigated. Broadly, this confirms that ranking according to SVM score is an effective way to direct manual annotators to the regions most likely to be productive.

However, not all of the protein-coding genes we identified ranked this well. In fact, during our survey of all lincRNAs, eight protein-coding genes were identified based on clusters with a best rank greater than 3000. Analysis of these cases identified two scenarios whereby protein-coding genes may have low PhyloCSF scores. First, the score can be lowered because of the loss of the gene in a sizable subclade, as this causes a gap in the underlying genome alignments. For example, *FAM240C* was apparently lost at the base of the rodent/lagomorph clade and was identified based on a cluster with a top rank of 22,742 (Supplemental Data S2). Second, although multispecies alignments aim to capture “1:1” orthology between genome sequences, they can be compromised by paralogy. Thus, *ETDA* and *ETDB* were identified as primate-specific duplications of a single-copy ancestral protein-coding gene, and it was apparent that the genome alignments producing their PhyloCSF signals were incorrect. We subsequently found evidence that certain high-ranking PCCRs were also based on alignments corrupted by paralogy, especially among the small cysteine and glycine repeat containing family members found in a cluster on Chromosome 2. In fact, local homology-based searching found three additional novel protein-coding genes within this cluster supported by PCCRs beyond the rankings studied here (*SCYGR1*, *SCYGR5*, and *SCYGR7*), and also identified *ETDC* as an additional paralog to *ETDA* and *ETDB*. These genes are included in Supplemental Data S2.

### Seventy protein-coding genes are new discoveries

We believe that 70 of the 144 protein-coding genes added to GENCODE in this study are new discoveries, in that they were not considered to be coding loci in human before they were annotated and made publicly available by GENCODE (the sources we searched in order to come to this conclusion are listed in Supplemental Methods section “Assessing the novelty of annotations”). We found that 61 of the 144 genes existed before this study in either the RefSeq or UniProt catalogs or were previously characterized as ORFs by Mackowiak et al. (2015) based on their usage of PhyloCSF. However, it appears that 19 of these 61 genes have had their “correct” CDS resolved for the first time as part of this study. Next, as previously noted, we found that the CDSs identified within the 5′ UTRs of *MKKS* and *MIEF1* had already been reported in published studies (Akimoto et al. 2013; Andreev et al. 2015; Delcourt et al. 2018), although these findings had not propagated into any annotation catalogs. Finally, we rediscovered five out of the 16 protein-coding loci that we recently reported (Wright et al. 2016) based on a concurrent reanalysis of large “draft proteome” peptide data sets (Kim et al. 2014; Wilhelm et al. 2014) and all six loci from our analysis of testis data from the Chromosome-Centric Human Proteome Project (Supplemental Data S1, S2; Weisser et al. 2016).

Five of the 70 novel protein-coding genes were independently reported subsequent to our public release. *SMIM38* was reported as translated based on proteomics data (Ma et al. 2016), and three were experimentally characterized, namely *SPAAR* (Matsumoto et al. 2017), *STRIT1* (Nelson et al. 2016), and *MYMX* (Bi et al. 2017). We recognize that such experimental analyses will be important to confirm the functionality of all 144 protein-coding genes. Also, C12orf81 was independently included in the CHESS database based on an earlier GenBank prediction (Pertea et al. 2018). Finally, we note that *FAM240C*, *SMIM28*, and *AC138647.1* were annotated as protein coding in earlier versions of GENCODE and RefSeq. We included them as “new discoveries” because at the time of this study annotators in both groups had subsequently reconsidered all three loci to be noncoding. We emphasize that our classification of new discoveries refers to the time each gene was first made publicly available in GENCODE. Since that time, many of them have been incorporated into the RefSeq and UniProt catalogs, as well as other databases such as neXtProt and CHESS (Gaudet et al. 2017; Pertea et al. 2018).

Approximately half of these 70 genes have an ortholog with some form of prior gene annotation in another species, including 19 in mouse GENCODE, although with an incorrect translation in many cases (Supplemental Data S2). Following our comparative annotation, with the exception of certain paralogs discussed above, all except 15 of the total 144 human protein-coding genes now have annotated orthologs in mouse GENCODE; the missing cases apparently represent gene loss events. Furthermore, we found that at least 71 of the 144 arose before the mammalian radiation, and we were able to describe 15 zebrafish orthologs as part of the HAVANA/ZFIN annotation efforts (Howe et al. 2013).

### PhyloCSF finds additional CDS within known protein-coding genes

Although our main focus in this manuscript is on the set of protein-coding genes added to GENCODE, the majority (59%) of CDS base pairs added to GENCODE were in fact added to 236 previously annotated protein-coding genes. For 118 of these genes, the added CDS was a new discovery, in that it was not already present in the RefSeq or UniProt databases at the time it was made publicly available by GENCODE. An extreme example is the *RP1* locus, linked to retinitis pigmentosa, in which an additional transcript model containing 22 conserved novel coding exons was added to both the human and mouse gene sets. The bulk of these coding exons had been regarded by GENCODE and RefSeq as a separate protein-coding gene in human (LOC107984125), but our transcriptomic analysis indicates that these are not separate loci. Similarly, we were able to resolve the previously separated *BTBD8*/*KIAA1107* and *LCOR*/*C10orf12* gene pairs into single loci.

### PhyloCSF identifies pseudogenic regions

We added 169 pseudogenes to human GENCODE, according to the observation of nonpolymorphic truncating deletions, premature termination codons, or frame-disrupting changes in the human CDS in comparison to an inferred ancestral model (see Supplemental Methods section “Manual annotation overview”). Of these 169 pseudogenes, 149 appear to be new discoveries in that they were not included in the RefSeq catalog either. We also extended the structure of 24 previously annotated human pseudogenes and found evidence for “pseudoexons” within 32 protein-coding genes, that is, cases in which a portion of the ancestral CDS was lost within a gene that has apparently continued to encode a functional protein (Supplemental Data S1). Although 44 of the 169 pseudogenes are orthologs of ancestral protein-coding genes disabled in the human lineage (“unitary pseudogenes”), the other 125 are duplicative (“unprocessed”) pseudogenes, for which the PhyloCSF signal resulted from nonsyntenic alignment to protein-coding paralogs. The inclusion of these 44 increased the number of unitary pseudogenes in human GENCODE by almost a quarter (Supplemental Data S3). To our knowledge, 39 of these unitary pseudogenes were not found in other *human* databases, but 29 had protein-coding mouse orthologs recognized in either the GENCODE or RefSeq catalogs. We also added six mouse orthologs for these human loci, two of which are also unitary pseudogenes. One of these is the remnants of *crescent*, previously characterized in chicken (Pfeffer et al. 1997) and a recognized mammalian pseudogenization event (Kuraku and Kuratani 2011). The other four cases are mouse protein-coding genes that apparently represent new discoveries. For example, *Tmem274* has an ancient CDS; conservation may even extend beyond vertebrates, yet the pseudogenization appears unique to human (Fig. 2E). Meanwhile, *Pfn5* is a novel profilin-like protein-coding gene in mouse with a novel unitary pseudogene counterpart in human, *PFN5P* (Supplemental Fig. S3E). Studying the function of these genes in those species that have retained them could help us understand how their loss has affected the evolution of our species.

In certain cases, the protein-coding versus pseudogene decision was difficult, and Supplemental Data S2 highlights nine “edge cases” for which further experimental analysis will be especially important. These include pseudoexon cases and also genes in which the disruption to the ancestral CDS in human or mouse was relatively minor. It can be difficult to infer how the loss of CDS affects a protein-coding gene, as exemplified by *KIF25*, in which we found eight pseudoexons upstream of the previously annotated human CDS that are apparently not transcribed in higher primates despite showing vertebrate conservation, and yet, there is published evidence that the human locus produces a functional protein; we infer this must be a truncated molecule (Decarreau et al. 2017). Finally, we also recognize that certain reclassifications of lncRNAs as protein-coding genes would seem to contradict the findings of previous studies; this includes *TUNAR* (Lin et al. 2014) and *TINCR* (Kretz et al. 2013), both of which have ascribed noncoding functions. Their CDS are small, 48 aa and 87 aa, respectively, and yet, both are conserved beyond the mammalian order. In fact, we do not rule out the possibility that these loci function at both the protein and RNA levels.

### Proteomics data validates CDS annotations

The GENCODE proteomics pipeline provided additional support for six of the protein-coding genes that we did not already report in our parallel mass spectrometry–based protein-discovery efforts (Supplemental Data S2, S4; Weisser et al. 2016; Wright et al. 2016), including two of the 70 new discoveries. We also found support for CDS annotations added to 29 existing protein-coding genes (Supplemental Data S4). The GENCODE proteomics pipeline reprocesses the raw peptide spectral peptide data from Kim et al. (2014). This covers 30 tissues, allowing us to find, for example, peptide support for SMIM36 in retina to match the eye-specific transcription profile and peptide support for SMIM39 in frontal cortex to match the brain/central nervous system–specific expression profile. Nonetheless, our transcriptomic analysis indicates that many of the protein-coding genes are expressed in tissues from which peptide data are not yet available. Furthermore, as a result of our work, many of our 70 new discoveries now have corresponding entries in the neXtProt protein database (Gaudet et al. 2017), which aims to provide functional support for all human proteins. neXtProt protein sequences are taken from UniProt, which targets new GENCODE CDSs (such as our 70 new discoveries) for curation, and their mass spectrometry data are incorporated from PeptideAtlas (Desiere et al. 2006). We found that an additional seven of these genes currently have peptide support according to neXtProt/PeptideAtlas criteria (Supplemental Data S2), although these are less stringent and include samples from cancer cell lines. Finally, we used the SORFS.org database of ORFs under 100 aa predicted from a comprehensive set of RP studies (Olexiouk et al. 2017) to find evidence of translation for six of our 50 CDS matching this size criterion (Supplemental Data S2).

### PCCRs that did not support annotation

Many of the high-scoring PCCRs that did not correspond to ORFs and are presumed to be noncoding false positives overlapped predicted promoter and enhancer regions. In the former case, we believe this is because the high GC content and density of triples containing CpG at promoters (i.e., CpG islands) can result in codon frequency distributions similar to those of coding regions, and also because we used PhyloCSF's “fixed” option for branch lengths in the underlying phylogenetic tree, which is faster and more accurate than the “mle” option on single codons but is more sensitive to the level of sequence conservation. Thus, the elevated conservation typical of promoter and enhancer regions improves their fit to PhyloCSF's coding model of evolution, increasing their scores. Subsequently, we have found that the ranks of CpG island-associated false-positive PCCRs can be downgraded by running PhyloCSF with the “mle” option, which scales all branch lengths by a maximum-likelihood estimated factor and is far less sensitive to sequence conservation. However, the overall consequences of using the fixed versus mle options remain to be ascertained.

There are also 26 PCCR clusters that we consider highly likely to be genuine that did not yet lead to productive annotation (category “under investigation” in Supplemental Data S1). These include 12 protein-coding genes in which PCCRs suggest that translation initiates upstream of the annotated ATG initiation codon but no alternative upstream ATG was apparent. These PCCRs could represent upstream alternative splicing events that have not yet been captured in transcript libraries or perhaps show the usage of non-ATG initiation codons (Kearse and Wilusz 2017). We also found compelling evidence for translation events either within or overlapping with previously annotated coding exons of *POLG*, *PCNT*, *PLEKHM2*, *ASXL1*, and *ASXL2* in alternative reading frames. These cases remain difficult to interpret.

### Variation evidence supports recent protein-coding selection

Evidence from human nucleotide variation indicates that purifying selection at the amino acid level has continued to act on the newly added CDS, in aggregate, in the human population, as well as on the subset consisting of just the 70 novel coding genes. In particular, we found that variants in the new CDS show a strong bias to be synonymous if translated in the predicted reading frame (Supplemental Fig. S5A), and derived allele frequencies for nonsense variants are significantly lower than those of missense variants, which are in turn significantly lower than those of synonymous variants (Supplemental Fig. S5B,C).

### New annotations reveal 118 protein-altering GWAS variants

**Figure 3.**
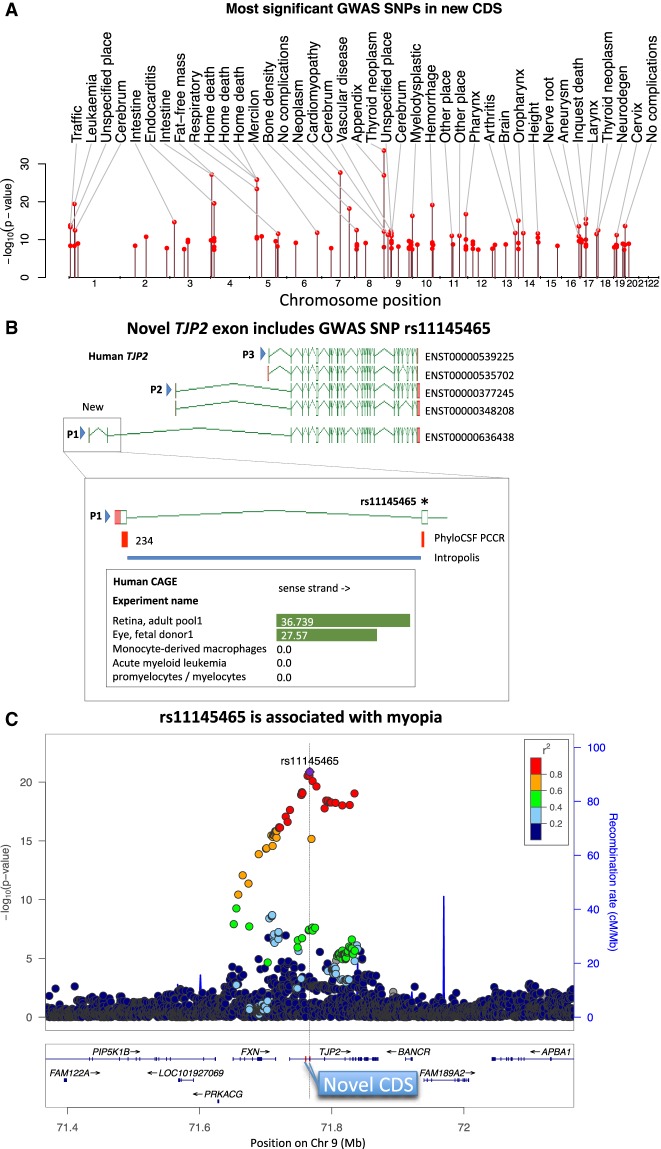
Protein-altering disease variants. (*A*) Chromosomal positions and strength of association for the 118 SNVs in newly annotated CDSs that were previously found to be significantly associated with diseases or other traits, with the trait abbreviation from Supplemental Data S5 listed for the 40 most significant associations. (*B*) Novel coding sequence added to human *TJP2* locus includes an eye disease–associated variant. Previous GENCODE annotation represented by models ENST00000539225, ENST00000535702, ENST00000377245, and ENST00000348208. Additional transcriptional complexity omitted for clarity. PhyloCSF PCCRs indicated the presence of two additional coding exons (dotted box and *inset*) that led to annotation of novel coding transcript model ENST00000636438, which lacks cDNA or EST support but whose intron is confidently supported by short read data in Intropolis (blue rectangle) mostly from a retinal study (Farkas et al. 2013), and whose TSS (P1) is supported by FANTOM5 CAGE data, limited to retina and eye (data from ZENBU browser, precisely redrawn for clarity; scores represent sequence read counts, with zeros for the next three experiments included for comparison). In contrast, TSSs P2 and P3 have negligible CAGE support for eye expression, with profiles dominated by monocyte and central nervous system expression. FANTOM5 CAGE also shows eye-specific expression for an equivalent mouse model added as part of this study, also supported by eye-experiment ESTs (e.g., BU505208.1). The second coding exon added to human GENCODE contains GWAS variant rs11145465, identified in a study of refractive error and myopia with a *P*-value of 7 × 10^−9^ (Verhoeven et al. 2013). In that study, the variant had been interpreted as noncoding based on RefSeq annotation, but it can now be reclassified as a missense mutation of an amino acid that is perfectly conserved in the mammal and avian clades. (*C*) Regional association plot for eye disease. All SNPs in an 800-kb window with their strength of association with refractive error and myopia in a more recent study (Tedja et al. 2018) show that rs11145465 has the strongest association. The positions of the novel coding exons of ENST00000636438 have been added in red.

An important application of gene annotation is to connect variants associated with disease via family studies or genome-wide association studies (GWAS) to changes in proteins. We searched the UK Biobank GWAS summary statistics and EBI GWAS catalogs for single-nucleotide variants (SNVs) within our new coding annotations that had previously been found to have genome-wide significant association with diseases or other traits. We identified 118 variants that affect the protein sequence, including one splice-disrupting variant, two nonsense variants, and 115 missense variants (Fig. 3A; Supplemental Data S5). Note that some variants might already have been classified as protein coding at the time of the GWAS because we have been releasing the updated annotations described here in GENCODE versions 24 through 28 and because some of the variants lie in regions previously classified as coding by RefSeq.

Recognition of these variants as protein disrupting may prove crucial in understanding the mechanism by which they affect disease. For example, a 2013 GWAS study found rs11145465 to be associated with refractive error and myopia, and had classified it as noncoding (Verhoeven et al. 2013; Tedja et al. 2018). However, we now recognize that it is a missense mutation in a previously unidentified protein-coding transcript of *TJP2* (Fig. 3B,C). This gene has been implicated in a wide range of diseases, including cancer, hearing loss, liver disease, and immune disorders (González-Mariscal et al. 2017). The novel coding transcript is expressed only in eye tissues (Fig. 3B), whereas the GENCODE transcripts described before this work show negligible expression in eye.

### Novel CDSs in other species

**Figure 4.**
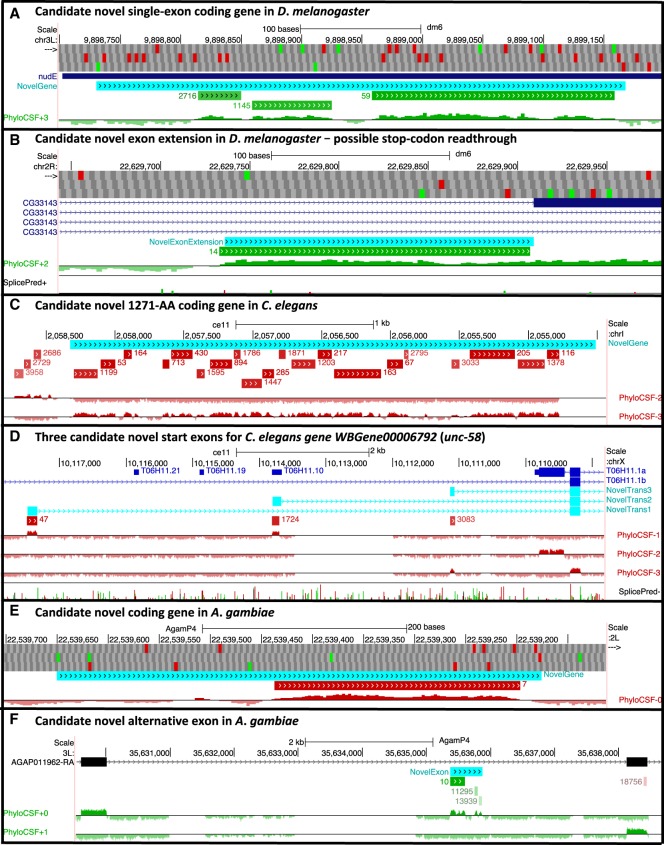
Potential novel CDSs in other species. Browser images show proposed novel CDSs (cyan) suggested by PCCRs (green/red for ± strand; rank next to region), smoothed PhyloCSF browser tracks, splice site predictions where useful (green donor, red acceptor, height indicating prediction strength), and ATG (green) and stop (red) codons. Supplemental Figure S6 has color-coded alignments for each example. (*A*) A cluster of three PCCRs in the 5′ UTR of *D*. *melanogaster nudE* suggest there is a single-exon novel protein-coding gene or an additional *nudE* cistron with ORF at positions 9898731–9899168. Although there is no PhyloCSF signal in the first 28 codons, the high frame conservation despite several indels provides ample evidence of purifying selection for protein-coding function. (*B*) A PCCR just 5′ of an exon of *D*. *melanogaster* transcript F of *CG33143* suggests that there is a novel coding transcript including an exon 173 nt longer than the annotated exon. This exon includes an in-frame TAG stop codon, suggesting translational stop codon readthrough. We have previously estimated that ∼6% of *D*. *melanogaster* genes undergo stop codon readthrough (Jungreis et al. 2016). The stop codon is perfectly conserved and is followed immediately by a cytosine residue, both of which are known correlates of readthrough. (*C*) A large cluster of PCCRs on the “−” strand of *C*. *elegans* Chromosome I suggests there is a 1271-amino-acid single-exon gene with ORF at positions 2054512–2058327. There is no alignment for a few codons on each end of the PhyloCSF signal, so to construct the putative ORF, we have extended the region 5′ to the nearest ATG and 3′ to the nearest stop codon. (*D*) Three PCCRs within an intron of *C*. *elegans* gene *WBGene00006792* (*unc-58*) shown on the “−” strand of Chromosome X suggest alternative start exons for that gene. The coding region of each of these putative exons begins with a perfectly conserved ATG and ends at a perfectly conserved GT having high splice-prediction score. All three end with a 1-nt partial codon, which allows them to splice to the next exon of transcript *T06H11.1b* while preserving the reading frame. (*E*) A PCCR in *A. gambiae* suggests that 22539177–22539650 on the “−” strand of Chromosome 2L is protein coding, forming either a novel gene or the first coding exon of the previously incompletely annotated gene *AGAP005849*. Subsequent curation confirmed the latter. Frame conservation provides strong evidence of coding function in the early portion of the putative transcript where the PhyloCSF signal is weak. (*F*) A cluster of three PCCRs in an intron of *A. gambiae* gene *AGAP011962* suggests an additional coding exon at positions 35635374–35635874 of Chromosome 3L, confirmed through subsequent curation to be part of a previously missed alternative transcript.

We have created PhyloCSF browser tracks and PCCR lists for chicken, fly (*D*. *melanogaster*), worm (*C*. *elegans*), and mosquito (*A*. *gambiae*). A cursory examination of top-ranked PCCRs in these lists suggests that implementing our complete workflow could prove useful for discovering hundreds of novel CDS and pseudogenes in those genomes. We describe a few examples from these species to indicate the potential value of such an effort (Fig. 4; Supplemental Fig. S6). These examples were identified from the alignments using the PhyloCSF signal, splice site predictions, and conservation of start codons, stop codons, splice sites, and reading frame, without reference to transcriptional data, so we cannot rule out that some of these are pseudogenes or that the true transcript models deviate from our predicted models.

Many of the best-ranked PCCRs in each of these species suggest novel pseudogenes (Supplemental Fig. S7), which is particularly notable because *D*. *melanogaster* and *A*. *gambiae* have a paucity of known pseudogenes.

We have also created PhyloCSF browser tracks and a PCCR list for the mouse genome. Our analysis of the human PCCR list has already resulted in many novel annotations in the mouse genome, and the mouse PCCR list could prove to be valuable for identifying novel annotations in regions of the mouse genome that have been lost in human. GENCODE plans to implement a full survey of mouse PCCRs.

## Discussion

We have presented the first whole-genome PhyloCSF resources for the human, mouse, chicken, *D. melanogaster*, *C. elegans*, and *A*. *gambiae* genomes and have shown the utility of the human resource and our workflow in finding hundreds of high-confidence novel CDS and pseudogenes within a genome that had already been intensely scrutinized. This analysis has several advantages over previous studies having similar goals. We have achieved high sensitivity by using PhyloCSF on the whole genome to find novel CDS that either fully or partially lie outside existing transcript catalogs. We have achieved high specificity by computationally filtering out identified sources of PhyloCSF false positives (including antisense signals, known pseudogenes, and low alignment branch length) and by manual examination of every candidate, retaining only those that were supported by both transcriptional and comparative data. Our integrated annotation workflow has allowed us to achieve more reliable and comprehensive results than could be achieved by either fully automatic or manual methods acting separately. In particular, although it is apparent that most PhyloCSF signals remaining after computational filtering are false positives, we have shown that our ranking algorithm is a highly efficient approach to isolate true positives. Meanwhile, our preview of the top-ranked PCCRs for *D. melanogaster*, *C. elegans*, and *A*. *gambiae* suggests that the deployment of a similar manual annotation–centered workflow guided by PCCRs could be a key step in completing the catalogs of conserved protein-coding genes for these species. A similar effort for the chicken genome is already underway (Vignal and Eory 2019).

Our whole-genome resources are already helping researchers investigating novel transcript sets to distinguish those with protein-coding potential without having to install and run PhyloCSF (Makarewich et al. 2018; Perry et al. 2018; Huang et al. 2019a,b; Kang et al. 2019; Lin et al. 2019; McCorkindale et al. 2019; van Heesch et al. 2019; Vignal and Eory 2019; Wang et al. 2019). Transcripts that do not overlap a PCCR or any annotated coding gene are unlikely to have conserved protein-coding function, whereas transcripts that overlap top-ranked PCCRs are the best candidates for translational potential. We recommend that no gene be considered protein coding based on PCCR overlap alone; rather, an overlap is the starting point for constructing a potential CDS. In this regard, CodAlignView is a valuable tool for exploring multispecies alignments for signals of coding potential (https://data.broadinstitute.org/compbio1/cav.php), and the PhyloCSF browser tracks may be especially useful for examining PCCRs in the context of transcriptomic data. Indeed, we stress the value of an integrated transcriptomic analysis: Many of our novel protein-coding genes previously existed as noncoding models that were inaccurately or incompletely described. Conversely, short-read transcriptomic data are not in themselves sufficient to identify protein-coding genes with high confidence, and even when the locus-level identification of coding potential is correct, we have found that the actual CDS predicted is commonly inaccurate. A confounding factor here is the existence of extensive alternative transcription within protein-coding genes. The proportion of this complexity that represents stochastic “noise” remains to be ascertained (Pertea et al. 2018; Wan and Larson 2018), and although it could be that only a minority of transcript isoforms are translated into mature proteins, this remains highly debated (Mudge et al. 2013; Blencowe 2017; Tress et al. 2017). In fact, we believe that PhyloCSF and our PCCR list have enormous potential both to discover additional *novel* protein-coding alternatively spliced transcripts in known genes and to distinguish those *known* transcripts that generate conserved protein products from those that do not (see Supplemental Fig. S2 for one example); our present work has only scratched the surface in this regard.

We recognize that not all novel protein-coding genes can be found by our workflow, and a brief survey of the 7% of previously annotated protein-coding genes that do not overlap a PhyloCSF Region found that many are recent paralogs lacking sufficient evolutionary history to produce a signal. We also reiterate that the fidelity of PhyloCSF is linked to the accuracy of the underlying genome alignments, and although “serendipitous” PhyloCSF signals resulting from paralogous alignments were of value to this study, we caution that this behavior cannot be relied upon. Furthermore, PhyloCSF confirms the *provenance* of a genomic region to be a protein-coding sequence, not whether it remains protein coding in a particular species. An examination of variation burden indicates that our novel CDSs, in aggregate, have continued to be subject to purifying selection at the amino acid level in the human population, but does not have adequate statistical power to show that each individual gene is still producing a functional protein. Showing that candidate CDS are not pseudogenic regions remains a judgement call until true confidence in the coding potential of a given gene can be obtained in the laboratory, ideally via single-gene studies. In the meantime, confidence in CDS annotation can be gained through the incorporation of orthologonal data sets. Although others have sought to discover or validate prospective CDSs using RP data sets (Bazzini et al. 2014; Mackowiak et al. 2015), our own experience is that these remain difficult to interpret in a biological context, certainly when the goal is to create “high-confidence” reference annotation (Mudge and Harrow 2016). However, we do not doubt the potential usefulness of RP data; indeed, we have shown that at least some ORFs initially suggested by RP are likely to be true proteins. Meanwhile, we and others have previously found novel CDSs using mass spectrometry (Slavoff et al. 2012; Kim et al. 2014; Wilhelm et al. 2014; Weisser et al. 2016; Wright et al. 2016). Our work here provides further demonstration of the value of this approach, and it has the potential to be extended in the future via “targeted” proteogenomics, for example, using synthetic peptides. Furthermore, GENCODE annotation is used by several projects seeking to provide catalogs of protein function, including neXtProt (via UniProt) and the Human Protein Atlas (Gaudet et al. 2017; Thul et al. 2017); we anticipate that such resources will in due course provide valuable insights into these genes based on experimental data.

Ultimately, experimental characterization of novel CDSs is vital, as gene annotation supports virtually all attempts to understand the mechanisms of human disease. Our preliminary work has shown that CDS discovery can shed light on disease-associated loci, and we hope that our reclassification of many disease-associated variants as protein altering will lead to further investigation of their mechanism of action and eventually to clinically beneficial consequences.

## Methods

### PhyloCSF

PhyloCSF software and parameters were obtained from GitHub (https://github.com/mlin/PhyloCSF [accessed August 28, 2014]). PhyloCSF was run using the “fixed” option on every codon in each frame on both strands of each chromosome and scaffold in the primary genome assembly. We used the “fixed” option because it is faster and, on single codons, more accurate than the “mle” option (though the “mle” option is more accurate on longer regions). Alignments used are specified in Supplemental Methods section “PhyloCSF and browser tracks.” The scores were smoothed using an HMM having four states, one representing coding regions and three representing noncoding regions. The emission of each codon is its PhyloCSF score. The ratio of the emissions probabilities for the coding and noncoding models is computed from the PhyloCSF score, because it represents the log-likelihood ratio of the alignment under the coding and noncoding models. The three noncoding states have identical emissions probabilities but different transition probabilities (they can only transition to coding) to better capture the multimodal distribution of gaps between same-frame coding exons. The emissions probabilities of the three states can be thought of as roughly capturing the gaps between a coding exon and the next coding exon on the same strand in the same genomic frame if they are consecutive exons in the same gene, are nonconsecutive exons in the same gene, or are in different genes. However, the algorithm does not actually use this information and instead uses expectation maximization to find the best approximation of this gap distribution as a mixture model of three exponential distributions.

The HMM defines a probability that each codon is protein coding, based on the PhyloCSF scores of that codon and nearby codons on the same strand in the same frame, without taking into account start codons, stop codons, or potential splice sites. The smoothed PhyloCSF browser tracks show the log-odds that each codon is in the coding state according to the HMM. PhyloCSF Regions are defined as the intervals in which the most likely path through the HMM is in the coding state.

PCCRs relative to a particular set of gene annotations were created as follows. All PhyloCSF Regions were compared to CDS and pseudogene annotations from the specified gene set, and those contained in annotated CDS regions in the same or antisense frame, or in annotated pseudogene regions in any frame or strand, were excluded. If only part of a region was contained in the annotated CDS or pseudogene, the region was trimmed to the unannotated portion. Regions shorter than nine codons were excluded.

We trained an SVM to distinguish PhyloCSF Regions that are more likely to be a novel coding region than antisense to a novel coding region, using as features the average PhyloCSF score per codon, the per-codon difference between the PhyloCSF score and the score in the antisense frame, and the length of the region. The length is relevant because antisense “ghost” regions tend to be shorter than true protein-coding regions. We trained the SVM using 10,000 randomly selected PhyloCSF Regions overlapping annotated CDSs in the same frame as positive examples, and an equal number overlapping annotated CDSs in the antisense frame as negative examples. We then excluded from the PCCRs set any regions that our antisense SVM scored below 0.3, a threshold chosen so as to keep almost all of our positive training examples (99%), while excluding most of our negative training examples (94%) (Supplemental Fig. S1A).

We trained an SVM to distinguish the PCCRs most likely to be protein coding (Supplemental Fig. S1B) using four features, namely, the average PhyloCSF score per codon, the per-codon difference between the PhyloCSF score and the score in the antisense frame, the length of the region, and the branch length of the species in the local alignment of the region. These features were chosen because true protein-coding regions tend to have higher PhyloCSF score, a greater difference between PhyloCSF scores on the two strands, greater length, and greater alignment branch length than false positives (Supplemental Fig. S1C). We trained the SVM using 10,000 randomly selected PhyloCSF Regions overlapping annotated CDSs in the same frame as positive examples, and an equal number of regions that do not overlap any CDS annotations in the same frame or antisense frame or any pseudogene annotations in any frame on either strand as negative examples. We then ranked the PCCRs using the scores from this SVM. Both SVMs were trained using the “R” language svm function from the cran “e1071” package with default parameters (R Core Team 2017). To test whether the SVM performance statistics reported in Figure 1B were influenced by overfitting, we redid those calculations excluding the 10,000 training regions. There were only 60 known coding genes (0.3%) that overlapped at least one of the training regions but did not overlap any other PhyloCSF Regions, and excluding training regions when scoring known coding genes had a negligible effect on the results.

For each assembly, the annotation version used to compute PCCRs and whether the PhyloCSF scores used by the SVMs were the original “fixed” scores or scores recomputed using the “mle” option are reported in the Supplemental Methods section “PhyloCSF and browser tracks.” The counts reported in the Results section are from the hg38 human assembly using GENCODE v23.

### Annotation

To aid manual annotation, PCCRs were clustered based on 10-kb sliding windows; this was because novel coding regions are often found as multiple exons of the same gene. All annotation was produced manually according to the guidelines developed by the HAVANA group for the GENCODE/ENCODE projects (Harrow et al. 2012). A detailed annotation workflow is provided in Supplemental Methods section “Manual annotation overview.” Briefly, in addition to sequences from the GenBank repository, annotation was also supported by SLR-seq (Tilgner et al. 2015), capture-seq Pacific Biosciences (PacBio) data (Lagarde et al. 2017), and a vast collection of publicly available short-read RNA-seq data sets as processed by the Intropolis project (Nellore et al. 2016). Transcription start sites were annotated based on cap analysis of gene expression (CAGE) libraries generated by FANTOM (The FANTOM Consortium and the RIKEN PMI and CLST (DGT) 2014), and polyadenylation sites were identified using PolyA-seq data (Derti et al. 2012). Insights into tissue specificity were chiefly gained from the CAGE and Intropolis data sets. Comparative analysis was also performed on non-GENCODE genomes and transcriptomes. Potential orthologs were initially sought using BLASTP (Altschul et al. 1990) on the vertebrate protein database at NCBI (NCBI Resource Coordinators 2017) and examined in their genomic context using the UCSC (Casper et al. 2017) and Ensembl (Zerbino et al. 2018) Genome Browsers. Orthologs were also identified based on manual cross-species genome alignments. The accuracy of these provisional models was examined using whatever experimental data were available for that species. Multispecies protein alignments were created using Clustal Omega (Sievers et al. 2011). Additional scrutiny was applied to annotations that overlap transposons (Supplemental Methods section “Overlap of novel annotations with transposon sequences”). Transposon overlaps were found by comparing novel CDS to RepeatMasker regions (Smit et al. 2013) obtained from the UCSC Genome Browser (Casper et al. 2017), excluding regions of repeat class Low_complexity and Simple_repeat.

### Proteomics analysis

The raw data published by Kim et al. (2014) covering 30 tissues in 85 higher-energy collision dissociation (HCD) mass spectrometry experiments were downloaded from PRIDE (PXD000561, PXD002967) and converted to mzML format. These mzML spectra were searched using multiple search engines in a high-confidence OpenMS workflow as described by Wright et al. (2016) and Weisser et al. (2016). The spectra were searched against a sequence database composed of all GENCODE v27 CDS transcripts combined with PhyloCSF sequences; an equally sized decoy database generated using DecoyPyrat (Wright et al. 2016) was concatenated and used to control FDR. Peptides were filtered to a posterior error probability of less than 0.01 and required to be significant in multiple search engines; a minimum and maximum length of six and 30 amino acids, respectively, was set; a maximum of two missed cleavages were allowed; and certain modifications such as deamidation were filtered out. The final list of peptides were then manually inspected and curated against the PhyloCSF sequences and CDS.

### Human variation

Germline SNVs in the CDS portion of a newly annotated coding gene or of a previously annotated coding gene containing new CDS were obtained from Ensembl release 91. For analysis of purifying selection, only variants having the “MAF” and “MA” tags in the Ensembl VCF file were used. Variants associated with disease were found by searching for SNVs in new CDS or adjacent splice sites having *P*-value less than 5 × 10^−8^ in the EBI GWAS catalog and autosomes in the UK Biobank GWAS summary statistics for 2419 traits provided by the Neale lab. Additional details are in Supplemental Methods section “Human variation.”

## Data access

The PhyloCSF tracks for the hg38 human assembly generated using the 58-mammal alignments and the tracks for the hg19 (human), mm10 (mouse), galGal4 (chicken), dm6 (fly), and ce11 (worm) assemblies may be viewed in the UCSC (http://genome.ucsc.edu) or Ensembl (http://www.ensembl.org) genome browsers by loading the “PhyloCSF” public track hub. The URL for this hub is https://data.broadinstitute.org/compbio1/PhyloCSFtracks/trackHub/hub.txt.

The tracks for hg38 using the 100-vertebrate alignment are available at https://data.broadinstitute.org/compbio1/PhyloCSFtracks/trackHub_hg38_100/hub.txt.

The tracks for hg38 generated by lifting over scores generated in hg19 using the 29-mammal alignment are available at https://data.broadinstitute.org/compbio1/PhyloCSFtracks/trackHub_hg38_29/hub.txt.

An assembly hub for viewing PhyloCSF tracks for the AgamP4 mosquito assembly in the UCSC or VectorBase genome browsers is available at https://data.broadinstitute.org/compbio1/AssemblyHubs/AgamP4/hub.txt.

A repository has been created for spreadsheets containing the list of PCCRs for each species and annotation set, with pertinent information for each PCCR such as the PhyloCSF and SVM scores. It is our intention to add PCCR lists for additional species or newer annotations sets as they become available. The repository includes a README file that describes the spreadsheet fields. The PCCRs that were the primary focus of this study are those in PCCRs.H_sapiens.hg38.GENCODE23.txt.gz. The repository is available at https://data.broadinstitute.org/compbio1/PhyloCSF_Candidate_Coding_Regions.

All human annotations described in this study are included in GENCODE (www.gencodegenes.org) release v29, although most also appeared in earlier releases, beginning with v24. All of our new human protein-coding genes were made public in release v28 or earlier. All mouse protein-coding genes are in release M19 or earlier. All annotations were first publicly available via the GENCODE Annotation Updates trackhub, which is updated every 24 h (http://ftp.ebi.ac.uk/pub/databases/gencode/update_trackhub/hub.txt).

Scripts implementing the HMM, SVM, and splice-prediction algorithms are in Supplemental Code S1 and also in the public GitHub repository, https://github.com/iljungr/PhyloCSFCandidateCodingRegions.git. Also included are a README file containing step by step instructions for using these scripts and a script that works through examples.

## Supplementary Material

Supplemental Material
