# Supplementary material for: Discovery of high-confidence human protein-coding genes and exons by whole-genome PhyloCSF helps elucidate 118 GWAS loci
Source: Genome Res. 2019 Dec;29(12):2073–87. doi: 10.1101/gr.246462.118 (PMC6886504; doi:10.1101/gr.246462.118)
Supplement: Supplemental Material [file supp_29_12_2073__index.html]

Discovery of high-confidence human protein-coding genes and exons by whole-genome PhyloCSF helps elucidate 118 GWAS loci — Supplemental Material 

# Discovery of high-confidence human protein-coding genes and exons by whole-genome PhyloCSF helps elucidate 118 GWAS loci

## Supplemental Material

- Supplemental\_Data\_S1.txt
- Supplemental\_Data\_S2.txt
- Supplemental\_Data\_S3.txt
- Supplemental\_Data\_S4.txt
- Supplemental\_Data\_S5.txt
- Supplemental\_Data\_S6.txt
- Supplemental\_Code\_S1.zip
- Supplemental\_Material.pdf
