# Supplementary material for: Discovery of high-confidence human protein-coding genes and exons by whole-genome PhyloCSF helps elucidate 118 GWAS loci
Source: Genome Res. 2019 Dec;29(12):2073–87. doi: 10.1101/gr.246462.118 (PMC6886504; doi:10.1101/gr.246462.118)
Supplement: Supplemental Material [file supp_gr.246462.118_Supplemental_Material_.pdf]

Supplemental Materials for *Discovery of high-confidence human protein-coding genes and exons by whole-genome PhyloCSF helps elucidate 118 GWAS loci*

Supplemental Methods **2**
Supplemental annotation methods **2**
Manual annotation overview 2
Summary diagram for the workflow used in this study 3
Transcriptomics analysis 3
Comparative annotation 4
Overlap of novel annotations with transposon sequences 6
Assessing the novelty of annotations 7
Additional considerations for the annotation of PCCRs in other species 7
PhyloCSF and browser tracks **8**
Human variation **9**
Supplemental Data Formats **10**
Supplemental Data S1 .... **10**
Supplemental Data S2 .... **11**
Supplemental Data S3 .... **13**
Supplemental Data S4 .... **14**
Supplemental Data S5 .... **15**
Supplemental Data S6 .... **15**
Supplemental Tables **15**
Supplemental Table S1 .... **15**
Supplemental Figures **16**
Supplemental Fig. S1. PhyloCSF Support Vector Machines. **16**
S1A. ROC curve for antisense SVM 16
S1B. ROC curve for PCCR-ranking SVM 17
S1C. Density plots of features used by SVMs 18
Supplemental Fig. S2. PhyloCSF specifically distinguishes protein-coding conservation **19**
Supplemental Fig. S3. Additional novel genes **20**
S3A. CDS discovered on novel transcript within a previous lncRNA gene 22
S3B. CDS discovered within a pseudogene-overlapping transcript 23
S3C. CDS discovered within a previously unannotated region 23
S3D. Novel CDS identified within ALDOA 5' UTR 24
Supplemental Fig. S4. Multi-species protein alignments **25**
S4A. Alignment for *SMIM31*, Figure 2A 25
S4B. Alignment for *C10orf143*, Figure 2B 25
S4C. Alignment for *CCDC201*, Figure 2C 26

S4D. Alignment for *H2BE1*, Figure 2D 26
S4E. Alignment for *EDDM13*, Supplemental Fig. S3A 26
S4F. Alignment for *SMIM41*, Supplemental Fig. S3B 26
S4G. Alignment for *C1orf232*, Supplemental Fig. S3C 27
S4H. Alignment for ENSG00000285043, Supplemental Fig. S3D 27
S4I. Alignment for *PFN5P*, Supplemental Fig. S3E 27
Supplemental Fig. S5. Polymorphism evidence supports recent protein-coding selection. 28
Supplemental Fig. S6. Alignments of the candidate novel CDS in Figure 4 29
Legend 29
S6A. Candidate novel single-exon coding gene in *D. melanogaster* 30
S6B. Candidate novel exon extension in *D. melanogaster* – possible stop-codon readthrough .. 31
S6C. Candidate novel 1271-AA coding gene in *C. elegans* 32
S6D. Three candidate novel start exons for *C. elegans* gene *WBGene00006792* (*unc-58*) 35
S6E. Candidate novel first exon in *A. gambiae* 36
S6F. Candidate novel alternative exon in *A.* gambiae 37
Supplemental Fig. S7. Candidate pseudogenes in *D. melanogaster* and *A. gambiae* 38
S7A. Candidate novel unitary pseudogene in *D. melanogaster* 38
S7B. Candidate pseudogene in *A. gambiae* 39
References 39

Supplemental Methods

Supplemental annotation methods

Manual annotation overview

Gene annotation was performed in accordance with the guidelines used by the GENCODE project, which were originally developed for the ENCODE project. All relevant annotations were made by the HAVANA manual annotation group at the European Bioinformatics Institute, and became publicly available within the GENCODE / Ensembl gene sets following the next annotation freeze and genebuild process. However, annotations were also made available 24 hours after creation via the GENCODE annotation updates hub ftp.ebi.ac.uk/pub/databases/gencode/update_trackhub/hub.txt. Note that GENCODE provides the annotation for the Ensembl human and mouse genebuild, and the annotation can be downloaded from either the GENCODE web portal https://www.gencodegenes.org/ or the Ensembl web browser https://www.ensembl.org/. Further details on the genebuild process can be found at https://www.ensembl.org/info/genome/genebuild/genome_annotation.html, and https://www.gencodegenes.org/pages/faq.html, and within (Zerbino et al. 2018; Frankish et al. 2018).

HAVANA manual annotation is based around a set of consistently applied guidelines, developed by the HAVANA group for the GENCODE / ENCODE projects (Harrow et al. 2012), which are available here: <http://ftp.ebi.ac.uk/pub/databases/havana/havana/Guidelines/Guidelines_March_2016.pdf>. This process is centred on the creation of gene models via the manual interpretation of RNA

and protein sequences aligned to the genome sequence, using the Zmap / Otterlace bespoke in-house software developed at the Wellcome Trust Sanger Institute.

Summary diagram for the workflow used in this study

This diagram outlines the key analytical stages, evidence datasets, and search algorithms used to generate GENCODE annotation for this study. All aspects are discussed in detail in the following text.

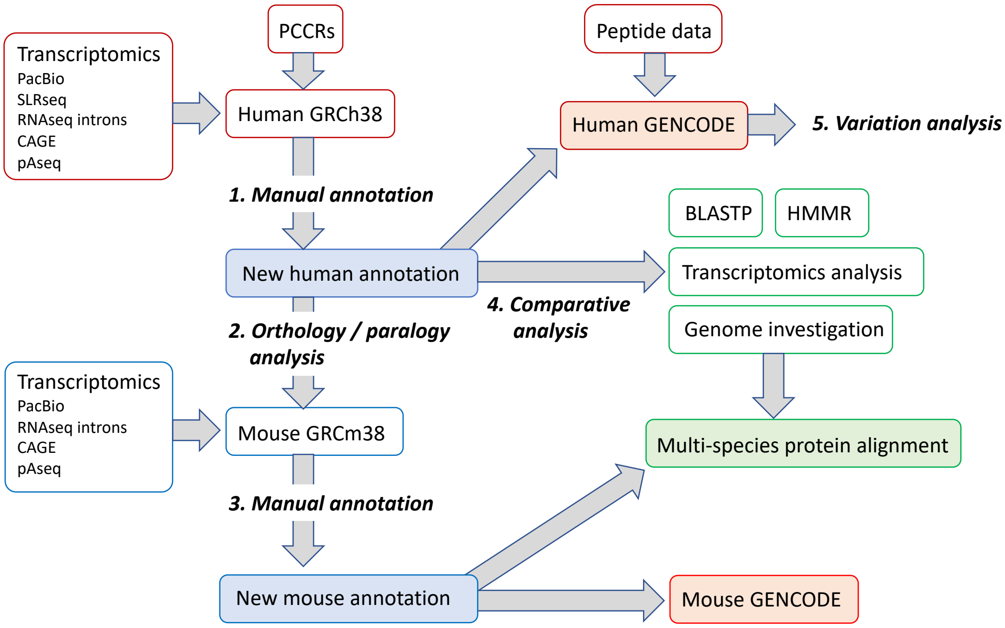

Transcriptomics analysis

Annotation reported in this study was carried out following the completion of the 'first pass' manual annotation of the entire human genome (hg38 / GRCh38) by the HAVANA group, where the core evidence sets used for annotation were mRNA, cDNA, and EST sequences in GenBank, and protein sequences from the SwissProt / UniProt project.

The present study utilised additional transcriptomics datasets, primarily to expand the GENCODE transcript catalog to incorporate sequences previously not recognised as transcribed, but also to provide insights into the specificity of transcript expression. The vast bulk of transcript annotations produced in this study were supported by RNA data from the following sources:

- PacBio data generated by GENCODE using a capture-seq methodology (Lagarde et al. 2017).
- Synthetic long-read sequencing (SLR-seq) data generated by Hilgner et al (Tilgner et al. 2015), based on brain tissues.
- Short-read RNA-seq data from a wide variety of external studies. Initially, these data were incorporated via the Intropolis resource, which has extracted a vast collection of RNA-seq data from SRA, reprocessed it according to consistent standards and presented a set of read-supported introns for public use (Nellore et al. 2016). A 'look-up'

table was created to allow the experimental provenance of the reads supporting a given intron to be judged, i.e. to provide information on tissue expression. However, Intropolis is not fully comprehensive, and additional RNA-seq data provided by the GTEx (GTEx Consortium 2013) and Blueprint projects (Adams et al. 2012) was manually assessed at the UCSC Genome Browser using the trackhub resources provided for each. Annotations were *not* based on transcript 'models' that were computationally assembled from short-read data (although the prior existence of such models proved useful in the analysis of non human or mouse genomes; see below). Such models were not used because they represent predicted combinations of splicing features and transcript endpoints, and may not capture *bona fide* transcripts.

- Cap Analysis of Gene Expression (CAGE) data provided by the FANTOM5 project (The FANTOM Consortium and the RIKEN PMI and CLST (DGT) et al. 2014). These were used to examine transcript start sites, and also the specificity of transcript expression. The datasets cover hundreds of different experiments, incorporating a wide range of normal somatic tissues.
- PolyAseq data provided by Derti et al. (Derti et al. 2012). These were used to help established transcript 3' ends, i.e. polyadenylation sites.

There was no requirement that a gene or transcript model must be supported by data from *all* of these sources, and it became apparent during this investigation that a subset of annotations have highly restricted expression. Thus, certain annotations lacked any long-read transcriptomics support (or cDNA or EST support), and were constructed by the careful manual analysis of RNA-seq-supported introns in concert with PhyloCSF data and comparative annotation, as described below.

Comparative annotation

The annotation of coding or pseudogenic sequences was initially inferred based on PCCRs as discussed in the main manuscript. However, all suggested annotations were also investigated in depth based on a process of manual comparative annotation. This process was seen to be essential in providing confidence in the annotation.

Comparative annotation proceeded according to the following workflow.

Human vs mouse

Firstly, manual HAVANA gene annotation equivalent to that performed on the human genome was also carried out on the mouse reference genome, version GRCm38/mm10. This was done to provide additional confidence in the human annotations, i.e. through the demonstration of conservation, but also to simultaneously improve the mouse GENCODE gene set. The progress of new mouse annotations into GENCODE and Ensembl occurs along essentially identical lines as described for human above, including visibility after 24 hours on the GENCODE annotation update trackhub.

The transcriptomics datasets used to support mouse annotation, in addition to nucleotide sequences in GenBank, were:

- PacBio data generated by GENCODE using a capture-seq methodology (Lagarde et al. 2017)
- Short-read RNA-seq data generated by the mouse ENCODE project (Mouse ENCODE Consortium et al. 2012). These had already been converted into read-supported introns by the Ensembl project. As for human, these data were *not* interpreted based on

computationally assembled short-read transcript models.

- CAGE data provided by the FANTOM5 project (The FANTOM Consortium and the RIKEN PMI and CLST (DGT) et al. 2014).
- PolyAseq data provided by Derti et al (Derti et al. 2012).

Orthologous mouse annotation was attempted for all prospective human annotations, i.e. for both new genes and new transcript models within existing genes, for both protein-coding genes and pseudogenes. The first stage was to establish gene orthology between the two species. For extensions to ‘known’ genes -- i.e. genes that were already recognised as functional protein-coding genes -- this was straightforward based on the existing Ensembl gene catalogs (which contribute to the Ensembl Compara gene homology resource), as well as EntrezGene. For prospective newly identified protein-coding genes and pseudogenes, human and mouse gene orthology was initially extrapolated through an investigation of whole-genome alignments. However, the accuracy of these alignments was manually assessed in all cases. Gene synteny between human and mouse was established manually, i.e. by a consideration of gene order. Care was also taken to identify potential paralogous sequence relationships between human and mouse that may have confounded the alignments. This was found to have occurred in numerous cases, and we emphasise the importance of manual analysis in the demonstration of ‘true’ orthologous gene relationships. This is especially true for the establishment of orthology across more distant evolutionary relationships, as discussed below, and the analysis of genome sequences of poorer quality than the mouse and human reference assemblies can provide additional complications.

Once gene annotation had been performed on the mouse genome, the prospective human genes or transcripts were manually compared against existing or new mouse counterparts. Transcript sequences were aligned using in-house tools, taking special care to manually assess splice site equivalency, and protein alignments were performed using Clustal Omega.

Analysis of other vertebrate genomes

Next, comparative analysis was performed on additional vertebrate species. The major focus was on species whose genome sequences are included in the whole-genome alignments. The phrase ‘comparative *analysis’* is used here because this process did not result in the creation of annotations in public catalogs, as for human and mouse GENCODE. An exception is the zebrafish genome, where annotations could be created for certain genes as part of the ZFIN project, to which HAVANA previously contributed.

Vertebrate comparative analysis occurred along two lines, which were ultimately convergent. Firstly, for prospective novel human protein-coding genes or pseudogenes, attempts were made to find existing CDS annotations in other vertebrate species. This was done by NCBI BLASTP analysis of the full non-redundant protein sequences collection held by GenBank (Altschul et al. 1990), and also by HMMER analysis of the full set of Ensembl vertebrate genome annotations (Potter et al. 2018). Both searches were required as the Ensembl databases do not incorporate RefSeq annotations, while the NCBI search does not analyse Ensembl annotations; however both searches do analyse the SwissProt / UniProt catalogs. Furthermore, GenBank -- unlike Ensembl -- also includes protein sequences from additional sources.

Secondly, vertebrate genome analysis was performed according to the same principles described for human and mouse manual annotation, although by necessity according to more basic criteria. Thus, gene orthology was initially suggested based on the whole-genome alignments, before being manually investigated in more detail. Transcriptomics resources were utilised according to availability, and knowledge of availability informed the choice of species

genomes to initially focus on (as did knowledge of the quality of genome sequences). We note the particular value to this study of the RNA-seq-based PLAR models generated and made publicly available by Hezroni et al (Hezroni et al. 2015), which cover 16 vertebrate genomes.

Critically, where existing protein sequences were found for other vertebrate genomes based on database searching, these sequences were reanalysed within the context of the genome of that species. Thus, translations that were initially provided by (e.g.) RefSeq or Ensembl were analysed in more depth using genome browsers, especially by a comparison against transcriptomics data for that species. As noted in the main manuscript, we were frequently found that the translations provided for these genes by RefSeq and Ensembl were not fully accurate, and we could manually extrapolate the 'true' full-length CDS as part of this process. We also caution that GenBank contains 'low quality' predictions based on the RefSeq computational pipeline for other vertebrate species. These can apparently occur when protein sequences are extrapolated from other vertebrate genomes based on comparisons against the gene annotation catalogs of reference genomes like human and mouse. In these predictions, the protein or CDS is artificially 'fixed' to provide an intact translation that matches that found in other species. While this process can presumably provide 'true' translations from species genomes that are 'broken' by genome sequencing errors, it can also mask genuine pseudogenisation events, and -- in our experience -- even provide spurious translations for loci that have no provenance as protein-coding sequence.

Ultimately, manual comparative analysis continued until we were satisfied that a thorough and (as far as possible) accurate evolutionary 'stratigraphy' had been established for each protein-coding gene or pseudogene. In other words, until we were confident that the provenance of each gene could be established within the vertebrate phylogeny, as demonstrated by the generation of a comprehensive multi-species protein alignment that satisfied the evolutionary principles of parsimony. This last point is important: in comparative annotation it is vital that arguments are not based only on the consideration of a small number of genome sequences that support the original supposition, i.e. that a sequence is protein-coding.

Overlap of novel annotations with transposon sequences

As part of the human manual annotation process, we checked for overlap with transposons. While it is well established that ancient transposons can contribute to the human proteome -- both by adding novel coding exons and by creating novel coding genes (Riordan and Dupuy 2013; Hayward et al. 2013; Sorek 2007) -- more recently inserted transposons can also be a source of false positive protein-coding annotations (Abascal et al. 2018). We found that 27 of our newly annotated protein-coding genes and 17 new CDS in previously annotated protein-coding genes contained at least one base pair of overlap with a transposon identified by RepeatMasker (Smit et al. 2013). However, in 21 cases the overlap was limited to an alternative transcript rather than the PhyloCSF-supported transcript listed in Supplemental Data S2, while for 18 cases the overlap was either a small fraction of the new CDS, was in a transposon call that could be reappraised as a false-positive, or was in the uncertain region near the edge of the transposon prediction. In the five remaining cases our comparative analysis verified that the transposon insertion occurred near the base of the mammalian radiation or earlier, and we are thus confident that these newly annotated CDS represent genuine 'domestication events' in the human proteome. For example, 100% of the novel CDS within *MED25* results from a DNA / hAT-Charlie transposon that added alternatively spliced exons to a gene that is conserved in vertebrates, and 69.4% of novel gene *PNMA8C* overlaps an LTR / Gypsy element. This gene is found across the mammal clade and is a paraneoplastic antigen-like protein, a family derived from Gypsy-like transposons with the CDS being co-opted from a functional ORF within the

transposon.

Assessing the novelty of annotations

We use the term 'novel' or 'new discovery' to describe a coding gene, coding sequence, or pseudogene that, at the time it was made publicly available by GENCODE, was not considered to be coding or, respectively, pseudogenic in the species under consideration in any of the major gene catalogs, or, as far as we could determine, in the peer-reviewed literature.

More specifically, we considered protein-coding genes added to human GENCODE as part of this work to be 'novel' if they satisfied the following criteria: (1) a corresponding protein-coding gene could not be found in the RefSeq database at the time the GENCODE annotation became publicly available (for most annotations this was RefSeq release 107); (2) the novel CDS did not have a counterpart in the UniProt / SwissProt database at that time; (3) an equivalent human protein-coding gene model could not be identified in the GenBank repository; (4) the protein-coding gene had not been identified in earlier published 'gene discovery' efforts that we were aware of (a comparison against the efforts of Mackowiak et al is included in Supplemental Data S2). We considered pseudogenes and newly annotated CDS in known protein-coding genes added to human GENCODE, or any annotations added to mouse GENCODE, to be 'novel' if they were not in the RefSeq or UniProt catalogs at the time the annotation become publicly available.

Note that GENCODE makes novel annotations publicly available every 24 hours prior to incorporation into a full release, using the GENCODE Annotation Updates track hub available at the Ensembl and UCSC Genome Browsers. (http://ftp.ebi.ac.uk/pub/databases/gencode/update_trackhub/hub.txt).

As pointed out in the Introduction of the main manuscript, by 'novel' we do not mean '*de novo*'. While these genes have not been previously annotated in *human*, in many cases they have orthologs that were identified by gene annotation efforts or experimental publications in other species, or are paralogs of genes that are already known in human.

Additional considerations for the annotation of PCCRs in other species

Broadly, we envisage that the PCCR sets created for *D. melanogaster*, *A. gambiae*, and *C. elegans* could be utilised according to a similar workflow to that presented for human; specifically, based on a manual approach that systematically examines PCCRs sorted according to SVM score. Each of these three organisms represents a 'model species', and as a result each has an appreciable amount of corresponding experimental datasets to support annotation, e.g. transcriptomics datasets. However, it is currently not clear whether the stratification of annotation outcomes presented for the human analysis will also be borne out in these species. There are several reasons for this, and these issues should also be considered by users considering the creation of whole-genome PhyloCSF datasets for additional genomes.

(1) The 'discoverability' of novel features depends on the extent and quality of gene annotation already available for a species. Obviously, if a genome has a relative paucity of quality annotation, then there will be more novel features to discover. The *D. melanogaster* and *C. elegans* genomes have been manually examined for many years by the respective FlyBase and WormBase projects. In contrast, the gene annotation of *A. gambiae* originates from combinations of automated annotation pipelines from Celera and Ensembl, later incorporating Genewise-based updates, and subsequently improved with ongoing VectorBase and community

curation.

(2) Discoverability will relate to the ‘biology’ of a given genome, specifically (a) the number of protein-coding genes, (b) the complexity of protein-coding genes, including CDS size and extent of alternative splicing, and (c) the size of the pseudogene compliment.

(3) The quality of a genome sequence can affect the fidelity of PhyloCSF signals, as well as the accuracy of existing gene annotation. Errors in a genome sequence -- e.g. incorrect bases, sequence insertions, sequence gaps etc -- can disrupt CDS, and our experience in the Ensembl project is that disruptions often incorrectly manifest as pseudogenic regions during computational annotation. Thus, we recommend that apparent pseudogenic regions identified by PCCRs in the three invertebrate genomes are re-evaluated in the context of genome sequencing information, e.g. the presence of known assembly problems. In our experience, variation datasets also provide an excellent resource for identifying unrecognised genome errors. Conversely, we note that the analysis may in fact identify novel variants, i.e. linked to polymorphic gene / pseudogene loci.

(4) We suspect that the quality of transposon annotations available for a genome may also have consequences for the generation of PCCRs. Pertinently, certain classes of transposons contain open reading frames, and if these are not ‘masked’ it is plausible they will manifest as PCCRs (presumably due to the codon frequency component of PhyloCSF as opposed to the interspecies-substitution component).

PhyloCSF and browser tracks

Multispecies alignments used for PhyloCSF were obtained from the UCSC Genome Browser (Casper et al. 2017), except for the *Anopheles* alignments, which were generated as described previously (Neafsey et al. 2015; Jungreis et al. 2016). Alignments and PhyloCSF parameter sets are summarized in the table below.

Species	Assembly	PhyloCSF Parameter Sets	Alignments
Human	hg19	29mammals	29 mammal subset of 46 vertebrates
Human	hg38	29mammals	Liftover from hg19 + special 29 mammal subset of 100 vertebrates (see below)
Human	hg38	58mammals	58 placental mammal subset of 100 vertebrates
Human	hg38	100vertebrates	100 vertebrates
Mouse	mm10	29mammals	29 mammal subset of the 60 vertebrates
Chicken	galGal4	49birds	49 sauropsids
Drosophila melanogaster (fly)	dm6	23flies	23 *Drosophila* subset of the 27 insects
Caenorhabditis elegans (worm)	ce11	ce11	8 *Caenorhabditis* subset of 26 worms
AgamP4 *Anopheles gambiae* PEST strain (mosquito)	AgamP4	21mosquitoes	21 *Anopheles*

We began working with the hg38 / GRCh38 human assembly before whole-genome alignments were available for that assembly, so scores for most codons were obtained by using *liftover* to find the corresponding codon in hg19, and then using the hg19 score of the resulting codon. When the 100-way vertebrates hg38 assembly became available, we added scores for codons that did not liftover to hg19 using a 29-mammal subset of the 100-vertebrates hg38 alignment, consisting of the species `Human`, `Chimp`, `Rhesus`, `Bushbaby`, `Mouse`, `Rat`, `Squirrel`, `Rabbit`, `Pika`, `Alpaca`, `Dolphin`, `Cow`, `Horse`, `Cat`, `Dog`, `Microbat`, `Megabat`, `Hedgehog`, `Shrew`, `Elephant`, `Tenrec`, `Armadillo`, `Chinese_tree_shrew`, `Guinea_pig`, `Marmoset`, `Star_nosed_mole`, `Manatee`, `Brush_tailed_rat`, and `Chinese_hamster`, and then dividing the score by 0.97 to make it as comparable to the lifted-over hg19 scores as possible. Subsequently, we also computed scores for every codon in hg38 directly using the 100-vertebrates alignment and, separately, using the 58-placental-mammals subset of the 100-vertebrates alignment.

Splice site predictions shown in the browser tracks were computed using the maximum entropy method (Yeo and Burge 2004), trained on splice sites from *D. melanogaster* for dm6, *C. elegans* for ce11, *A. gambiae* for AgamP4, and human for the other assemblies.

We computed PhyloCSF Candidate Coding Regions for hg19 (human) using GENCODE v19, for mm10 (mouse) using GENCODE version M5, for galGal4 (chicken) using Ensembl version 82, for dm6 (*D. melanogaster*) using version 6.15 from www.flybase.org (Gramates et al. 2017), for ce11 (*C. elegans*) using version WS259 from www.wormbase.org (Lee et al. 2018), and for AgamP4 (*A. gambiae*) using version 4.8 from www.vectorbase.org (Giraldo-Calderón et al. 2015). We computed PhyloCSF Candidate Coding Regions for the hg38 human assembly using GENCODE v23, and those are the ones whose counts are reported in the Results section. We have also computed PhyloCSF Candidate Coding Region lists relative to later GENCODE versions for use by the community.

For the hg38 human assembly, we computed three sets of regions, using:

1. the liftover PhyloCSF scores from hg19 based on the 29 mammals alignment,
2. the scores computed in hg38 using the 58-mammals alignment, and
3. scores computed using the 100-vertebrates alignment.

In each case, we computed three sets of rankings (for a total of nine region-list/ranking combinations) by using the original “fixed” PhyloCSF score for each region and scores recomputed for each region using the “mle” option with the 58-mammals or 100-vertebrates alignments. Most of our work was done using the 29-mammals regions with the original “fixed” scores, but we also investigated the lowest-ranking regions from each of the other eight combinations on an *ad-hoc* basis, which led to several additional new annotations, including five protein-coding genes labeled ‘re-ranking’ in Supplemental Data S2. For dm6, ce11, and AgamP4, we only computed rankings using scores recomputed using the “mle” option.

Human variation

Germline single-nucleotide variants in the CDS portion of a newly annotated coding gene or of a previously annotated coding gene containing new CDS were obtained from Ensembl release 91. Previously annotated coding genes containing new CDS are listed in the “Notes” field of lines in Supplemental Data S1 having category “coding exon(s)”. The new CDS in these genes refers to any genomic intervals in these genes that were annotated as coding in GENCODE v28 but not annotated as coding in GENCODE v23. Derived alleles were determined using the hg38 100-vertebrates alignment. The sample of previously annotated CDS used for comparison consisted of the previously annotated CDS in the coding genes containing new CDS.

Variants associated with disease were found by searching for SNVs in newly annotated CDS or adjacent splice sites having p-value less than 5×10^{-8} in the EBI GWAS catalog (MacArthur et al. 2017), accessed through the *gwascat* R package (http://bioconductor.org/packages/gwascat/, image version dated August 3, 2015), and autosomes in the UK Biobank GWAS summary statistics for 2419 traits provided by the Neale lab (http://pheweb.sph.umich.edu:5000/about, accessed December 20, 2017). One SNP, rs576793567, was excluded because it is in a CDS that was added to GENCODE for reasons unrelated to this project. Variants shown in Figure 3C and corresponding p-values are from the genome-wide meta-analyses conducted in (Tedja et al. 2018). Linkage disequilibrium was based on the EUR population in the 1000 Genomes Project (Nov 2014). Chromosomal coordinates and annotations shown in Figure 3C are from GENCODE v19 in the hg19 assembly.

The CDSs added to eight known genes -- *TBC1D30, LMTK3, FAM83H, CRYBG1, RHOT1, PLEKHG4B, NEDD4L*, and *SPTBN2* -- were either discovered or reclassified as coding after the variation analysis in this study had already commenced. These annotations are thus included in the total gene feature counts presented in the main text, although not in the variation calculations. Subsequently, we observed that these CDSs do not overlap any GWAS variants in the EBI catalog, or disease-linked variants in ClinVar.

Supplemental Data Formats

Supplemental Data S1-S6 are tab-delimited text files that can be viewed in a spreadsheet application or accessed programmatically. The fields are described below.

Supplemental Data S1

All PCCRs that were subjected to manual analysis. Each row corresponds to a cluster of one or more PCCRs. All genome coordinates are from the hg38 assembly.

- 'PhyloCSF start' and 'PhyloCSF end' list, respectively, the first and last genomic coordinate of the 5' and 3' PCCR in a cluster.
- The number of PCCRs that make up a cluster is also listed, alongside the coordinates of the PCCRs in the cluster (the 'PhyloCSF region ID'). It is possible that not all PCCRs within a cluster that led to productive annotation were individually informative. Meanwhile, certain clusters were found to elucidate multiple separate features, and so row counts extrapolated from this file do not precisely equate to the number of protein-coding gene and pseudogene identifications described in the main text.
- 'Top rank' lists the rank of the best (i.e. lowest ranked) PCCR in a cluster. This was based on the set of 29-mammals regions built on hg19 using the 'fixed' option and 'lifted' onto hg38, with the exception of the nine clusters listed as 're-ranking' in column K (see below), whose ranks were based on the set of regions built on hg38 using the 58-mammals alignment and ranked using the 'mle' option, as discussed in the "PhyloCSF and browser tracks" section of Supplemental Methods.
- CpG island overlaps were defined in-house; 'yes' indicates that at least one PCCR in the cluster has a coordinates-based overlap with a CpG prediction.
- Brief notes are provided on the interpretation of the PCCR cluster, as deduced by manual annotation.
    - 'Putative regulatory region' is used to denote clusters that did not lead to productive annotation and potentially represent genomic elements in either promoter or enhancer regions. Such manual interpretations were based on the resources provided by the Ensembl Regulatory Build.
    - Meanwhile, clusters were considered 'potentially spurious' when they did not

correspond to prospective open reading frames, and did not appear to represent regulatory regions based on current data.

- The ‘Category’ column is defined as follows
  - Definitions for the annotation categories ‘not annotated’, ‘coding exon(s)’, ‘protein-coding gene’, ‘pseudogene’, ‘pseudo-exon’ and ‘pseudogene extension’ are described in the main text. For pseudogene categories, if an HGNC gene symbol listed in the Notes column is placed in parentheses, this indicates that this symbol is for the inferred parent gene (for processed and unprocessed pseudogenes) and not the pseudogene locus itself. Official pseudogene symbols are listed without parentheses, while Ensembl IDs (words beginning with ‘ENSG’) are only listed for the pseudogene itself. Note that in a small number of cases, the PCCR cluster was seen to include more than one separate locus (‘shared cluster’).
  - ‘immunoglobulin segment’ describes those PCCR clusters that overlap a known immunoglobulin locus in GENCODE, which are categorised separately to protein-coding genes. These entries were removed from further analysis.
  - ‘known assembly issue’ describes a small number of cases where the coding potential of a locus cannot currently be described in GENCODE because a known error in the genome sequences prevents annotation.
  - ‘redundant’ describes cases where the CDS or pseudogene annotation overlapped multiple clusters, and the annotation is described at a cluster row elsewhere in the sheet.
  - ‘under investigation’ describes clusters that we consider likely to have genuine evolutionary provenance as protein-coding regions, but that could not immediately be used to create CDS or pseudogene annotation according to our criteria. After the completion of this study, six of these clusters led to the annotation of protein-coding genes (category “under investigation; now made coding”) but they are not counted in the totals reported here.
- The ‘List’ column indicates the stage of the analysis in which the PCCR cluster was investigated, as described in the Results section:
  - ‘first 1000 PCCRs’ denotes the 658 clusters containing the top 1000 ranked PCCRs;
  - ‘lincRNA PCCR’ denotes clusters that overlapped GENCODE lincRNA genes;
  - ‘previously unannotated PCCR’ denotes clusters with a top rank of between 1001 and 2,200 that did not overlap any previously existing GENCODE category;
  - ‘re-ranking’ denotes clusters based on the set of 58-mammals regions built on hg38 that were investigated in an ad hoc manner.
- The ‘Novel?’ column defines whether the annotation was novel at the time it was made public, as defined in this document in the section ‘Assessing the novelty of annotations’. Novelty was only assessed for the protein-coding gene, coding exon and pseudogene categories.

Supplemental Data S2

All protein-coding genes added to GENCODE during this study.

- ‘Novelty’ describes our classification of the novelty of the gene annotation, as described in the ‘70 protein-coding genes are new discoveries’ section of the manuscript.
- The ‘HGNC gene symbol’ for each locus is provided by the HUGO Genome Nomenclature Committee (HGNC). This work is ongoing, and genes without an official name and symbol are instead noted by the generic clone-based symbol provided by

Ensembl.

- The CDS start and end coordinates are from the hg38 genome assembly, and list the first coordinate of the initiation codon and the last coordinate of the termination codon respectively.
- The ‘Top rank’ is the rank of the best (i.e. lowest ranked) PCCR in a cluster, based on the 29-mammals alignments.
- ‘List’ notes the stage of the analysis within which the locus was found, as per Supplemental Data S1; i.e. the analysis of the first 1000 top-ranked PCCRs signals (‘1000’), the analysis of lincRNAs (‘lincRNA’), or the analysis of additional regions up to rank 2,200 that previously had no GENCODE annotation at all (‘unannotated’). Alternatively, 4 CDS were identified as paralogs in the vicinity of a novel protein-coding gene, while 5 CDS were identified based on an ongoing effort to reappraise PhyloCSF ranking based on additional genome alignments (re-ranking).
- ‘Model status’ notes how the transcriptional annotation of the locus has changed since GENCODEv22, prior to its annotation or re-annotation during the course of this work. CDS were thus described variously as being on a ‘gene added’ to GENCODE, on an ‘existing transcript’ that did not require any modification to its length or intron / exon structure, on a ‘transcript extended’ that was modified to incorporate the entire CDS, or as a ‘spliceform added’ to GENCODE within an existing gene.
- The specific biotype of the model in GENCODE v22 is listed where annotation was previously found at the locus.
- Each gene is also listed by its ‘Human GENCODEv28 gene ID’ (an Ensembl gene ID), and the 'Human GENCODEv28 transcript ID’ is used to specify the model within that gene which contains the CDS reported in this file. Ensembl IDs became available once the initial manual annotations were incorporated in a GENCODE release; all human annotations were available in GENCODE v28, which is the annotation used for Ensembl release 92, except for transcript ENST00000637802 in *EDDM13*, which was included in v27 (as ENST00000637802), mistakenly removed in v28, and was reinstated in v29 (as ENST00000649256).
- ‘Conservation’ details the most distant evolutionary relationship across which an ortholog of the gene has been identified with confidence, and synteny maintained. This is essentially simplified to mammals, avians, xenopus, coelacanth, or vertebrates (i.e. the gene is found in all vertebrate genomes analysed, with a preservation of synteny). However, certain paralogs are noted as unique to human or primates. Furthermore, where a prospective ortholog has been identified without the conservation of synteny this is noted.
- Mouse Ensembl IDs are provided for GENCODE M18. An exception is gene ENSMUSG00000118462, added in the latter stages of this study and first available as an annotation model in M19. A ‘known coding gene’ in mouse was one that had been annotated in either RefSeq or mouse GENCODE prior to being rediscovered here. Thus, a ‘previously undescribed coding gene’ was created as part of this work, and had not apparently been described previously in any other annotation databases at the time the annotation was made.
- A non-human representative model is provided for all non-paralog cases. If the locus is conserved in mouse but not beyond mammals, the mouse ID in the previous column is considered sufficient. Other listings are variously RefSeq models, HAVANA zebrafish models, CDS predicted by the AUGUSTUS *in silico* annotation pipeline (Stanke et al. 2008), PLAR models based on transcript data including short-read data (Hezroni et al. 2015), or in a small number of cases genome coordinates for a manually identified CDS that does not have transcription evidence in that species. In many cases, the CDS provided by these third party annotations were seen to be not precisely correct, and

efforts were made to manually 'complete' the model by considering other experimental datasets.

- All human and mouse loci were subject to manual expression profiling based on short-read data, as described in the Methods and Supplemental Methods. The use of a variety of experimental approaches and datasets makes it difficult to infer accurate quantifications, and these data were therefore used simply to suggest expression patterns of potential interest for future investigations.
- Where applicable, 'Contemporary RefSeq annotation' lists the RefSeq model equivalent to the CDS transcript annotated as part of this work at the time these annotations were made. Certain RefSeq loci have since been updated, including cases where the update was made based on the sharing of the information presented here (see main text).
- 'Bicistronic?' notes if the transcription of the CDS is linked to that of an adjacent gene, i.e. based on the sharing of exons
- Further information is provided in the 'Comments' column, which more generally provides any pertinent observations regarding the CDS, for example in terms of its potential functionality. If a protein is regarded as 'uncharacterised' that means we are currently unable to provide any insights into its nature based on the work presented here (for example, we have not detected homology to other known proteins).
- 'Peptide support' lists whether the CDS has supporting peptides from mass spectrometry datasets: 'Kim et al' highlights cases where we identified these matches as part of the reanalysis of the 'draft proteome' datasets carried out in this study (see Results and Methods), while 'neXtProt' highlights cases where we subsequently found peptide support in the neXtProt database, as extrapolated from PeptideAtlas. Note that Ensembl proteins can rapidly propagate into UniProt via curation, while the proteins in neXtProt are derived from UniProt. Thus, the neXtProt entries corresponding to the 70 protein-coding genes we refer to as new discoveries were ultimately 'seeded' by our original annotations. Further information on the 'Kim et al' peptide support is presented in Supplemental Data S4, while neXtProt entries can be queried using the Ensembl gene ID at https://www.nextprot.org/. Following the publication of this work, we anticipate that additional peptide matches will accumulate in neXtProt.
- 'sorfs.orf' support highlights CDS that are listed in this repository of short ORFs previously identified in ribosome profiling assays (see main text); 'no' is listed only for those CDS that are theoretically discoverable by the criteria employed.
- 'Edge case?' highlights those loci that were annotated as protein-coding genes based on how we viewed the balance of probability, although without the same degree of confidence as for other loci (see main text).
- 'CDS sequence' provides the translation of the human protein-coding transcript specified by the 'Human GENCODEv28 transcript ID'. Note that unlisted alternative translations have been annotated in GENCODE for numerous genes in this file based on transcript evidence for alternative splicing.

Supplemental Data S3

Human unitary pseudogenes annotated in this study. Each row corresponds to a cluster of one or more PCCRs.

- The description of the first seven columns is provided in the legend to Supplemental Data S1 or S2, as are the descriptions for human and mouse gene IDs.
- 'GENCODEv22' lists the annotation status of the pseudogene in that previous release.
- The ID of the RefSeq model available at the time the pseudogene annotation was made, if any, is listed.
- 'Transcribed?' indicates if there is manually-appraised RNA evidence for the

transcription of the pseudogene.
- The official symbol of the mouse gene provided by the Mouse Genome Informatics institute is listed where available, as is the ID of the corresponding RefSeq annotation available at the time the mouse annotation was made.
- ‘Point of pseudogenisation’ notes the evolutionary clade with which pseudogenisation appears to be consistent based on genome alignments, e.g. ‘primates’ means that the locus appears to be a pseudogene (i.e. the ancestral CDS is disrupted) in all primate species with genome sequence in the 100-way vertebrate alignment resource at the UCSC Genome Browser.
- An apparent coding model described within an outgroup species of this clade is listed, as is the nature of the mutation event(s) leading to pseudogenisation (PTC: Premature Termination Codon, i.e. nonsense mutation).
- ‘Notes’ provides additional insights into the locus, including information on protein homology if available.

Supplemental Data S4

Peptide support for human CDS annotations.
- 'Peptide' indicates the sequence of a single peptide obtained from mass spectrometry (see Methods), with [J] representing either [I] or [L] as these amino acids are indistinguishable.
- 'GENCODE gene ID' and 'GENCODE transcript ID' are as listed in Supplemental Data S2.
- 'Tissues (no. PSMs)' lists the tissues in which peptides were found, and the number of times a peptide was found per tissue (Peptide Spectrum Matches; PSM).
- 'Total PSMs' provides the total peptide count.
- 'RNA expression notes' provides information on the RNA expression profile of the relevant transcript, based on contemporary transcriptomics data (see Methods). For new protein-coding genes, this information is duplicated from Supplemental Data S2. For new coding exon(s), information has been obtained relevant to the alternative transcription event, e.g. CAGE data for alternative first exons and RNA-seq intron data for alternative splicing (see also legend for Supplemental Data S2).
- ‘Notes’ indicates the nature of the transcript model containing the PhyloCSF Candidate Coding Region.
- 'Category' distinguishes new protein-coding genes from new coding exon(s).

Supplemental Data S5

GWAS variants in newly added CDS. This is a tab-delimited text file with information about the 118 protein-altering SNVs in new CDS for which significant associations with diseases or other traits were previously found. It includes the SNP id; chromosomal coordinates; reference and alternate nucleotides, codons, and amino acids; minor allele frequency; list of transcripts; containing genes; indication of the level of novelty (from Supplemental Data S2, plus 'New CDS in existing coding gene'); indication of whether it is in the CDS or (in one case) a splice site; indication of whether it is missense or nonsense; source GWAS catalog (UK Biobank or EBI); list of associated traits, with corresponding p-values and Z scores; and trait with strongest (i.e., lowest) p-value, with the abbreviation used in Figure 3A. For SNVs in new coding genes, we have duplicated some of the information about the gene from Supplemental Data S2 that might be helpful in analyzing the trait association, including human and mouse expression data.

Supplemental Data S6

Details of PCCRs in manually evaluated clusters. Information about each of the 4035 PCCRs in clusters that were manually evaluated, including PhyloCSF and SVM scores, position with respect to previously known annotations, and the result of manual annotation. These are the PCCRs included in the "PhyloCSF region ID" column of Supplemental Data S1. The columns are the same as those in the data files in the PCCR repository (described here: https://data.broadinstitute.org/compbio1/PhyloCSF_Candidate_Coding_Regions/README.html) with the addition of two columns, 'AnnotationCategory' and 'AnnotationNotes', that show the 'Category' and 'Notes' fields of Supplemental Data S1.

Supplemental Tables

Supplemental Table S1

TranscriptRegion	protein-coding gene	coding exon(s)	pseudogene	pseudo-exon	pseudogene extension	under investigation	immunoglobulin segment	known assembly issue	not annotated	redundant	Total
**Extension5p**	0	13	0	4	0	7	0	0	1	1	26
**Extension3p**	0	1	0	4	0	0	0	0	1	0	6
**ExtensionMid**	0	11	0	0	1	0	0	0	1	0	13
**CDS**	2	8	4	3	0	6	0	0	6	1	30
**UTR5**	8	42	5	10	2	4	0	0	3	6	80
**UTR3**	2	14	0	5	0	0	0	0	3	0	24
**NC**	86	87	36	4	21	4	38	36	3	60	375
**CDSintron**	3	84	4	11	0	1	0	0	8	6	117
**UTR5intron**	0	6	11	8	0	0	0	0	1	0	26
**UTR3intron**	0	0	0	0	0	0	0	0	0	0	0
**NCintron**	4	5	20	1	10	1	0	0	3	11	55
**AntiCDS**	0	0	0	0	0	0	0	0	23	0	23
**AntiUTR5**	0	0	0	0	0	0	0	0	3	0	3
**AntiUTR3**	0	0	0	0	0	0	0	0	1	0	1
**AntiNC**	2	0	0	0	0	0	0	0	1	2	5
**AntiCDSintron**	7	0	4	0	0	0	0	0	5	0	16
**AntiUTR5intron**	0	0	4	0	0	0	0	0	0	0	4
**AntiUTR3intron**	0	0	0	0	0	0	0	0	0	0	0
**AntiNCintron**	7	4	6	0	0	0	0	0	1	2	20
**Intergenic**	50	26	63	0	8	3	1	0	10	15	176
**Total**	171	301	157	50	42	26	39	36	74	104	1000

Counts of top 1000 PCCRs by transcript region and annotation type. Counts of PCCRs among the top-ranked 1000 that resulted in each kind of annotation ('Category' in Supplemental Data S1), broken down by transcript region (overlapping CDS, extension of CDS, UTRs, etc.) defined by the 'OverlapTypes' in Supplemental Data S6. If a PCCR has several overlap types, only the first one is counted. If a PCCR has no overlap types, it is counted as 'Intergenic'.

Supplemental Figures

Supplemental Fig. S1. PhyloCSF Support Vector Machines.

(A) ROC curve for the SVM used to distinguish PhyloCSF Regions overlapping protein-coding regions in the antisense frame from ones overlapping in the coding frame. We excluded from further consideration regions whose scores were less than 0.3, a threshold that admitted 99% of our positive training examples while excluding 94% of our negative training examples. **(B)** ROC curve for the SVM used to rank the PCCRs. **(C)** Density plots show values of the four features used by the SVMs, computed on PhyloCSF Regions overlapping protein-coding annotations in the coding frame (black), ones that overlap protein-coding annotations in the antisense frame (red), and ones that do not overlap protein-coding or pseudogene annotations in any frame on either strand (green). Protein-coding overlapping regions were distinguished from antisense overlapping regions using the score per codon, the per-codon difference between the score on the two strands, and the length of the region in codons. The SVM used to rank the PCCRs used these features plus the relative branch length of the local alignment.

S1A. ROC curve for antisense SVM

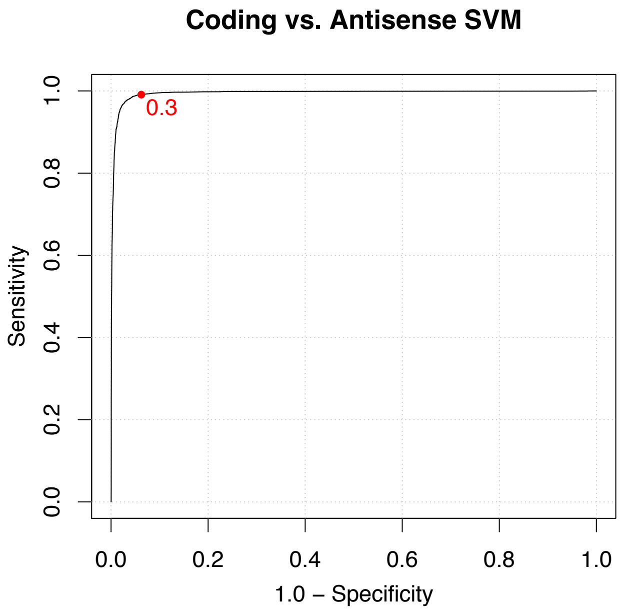

S1B. ROC curve for PCCR-ranking SVM

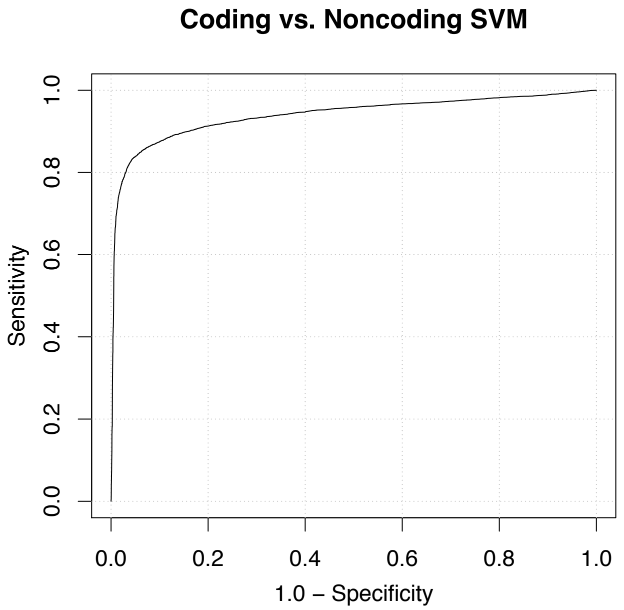

S1C. Density plots of features used by SVMs

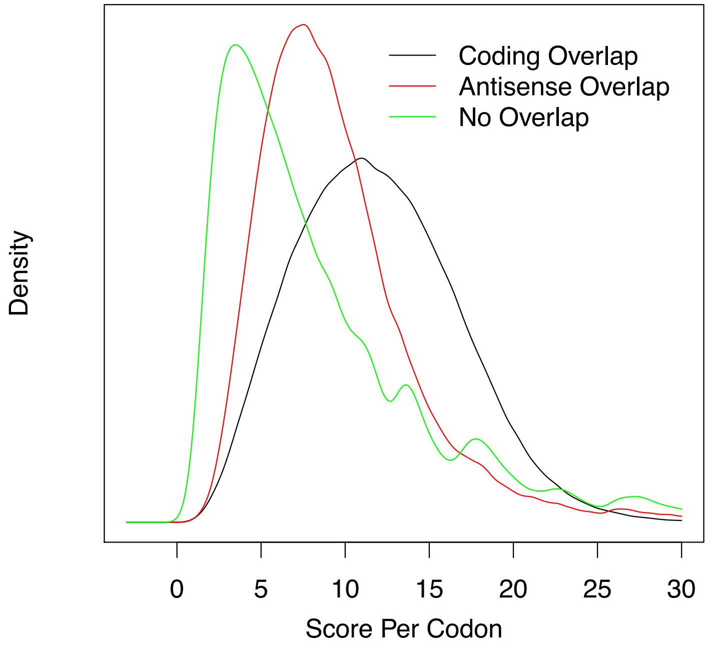

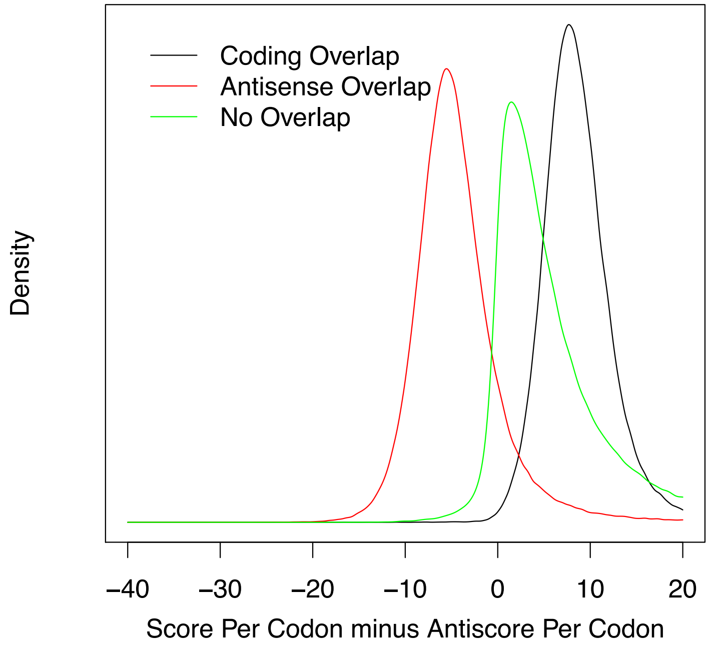

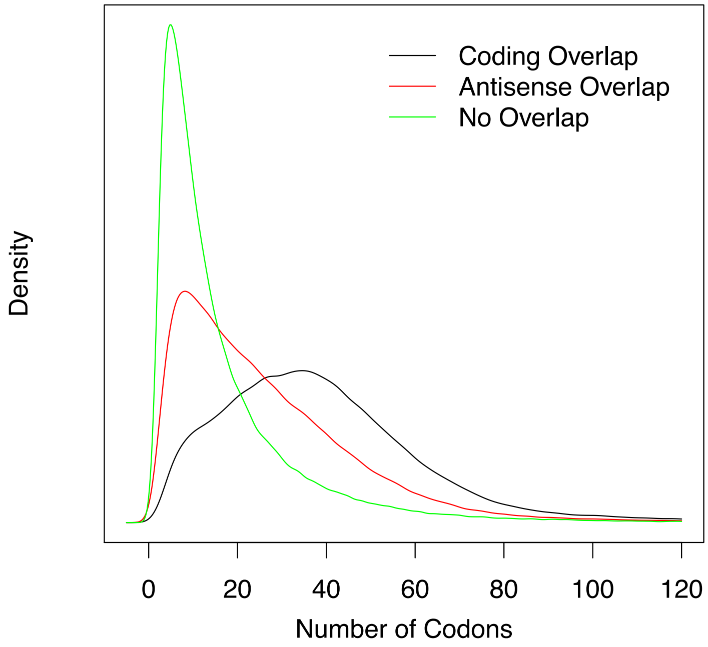

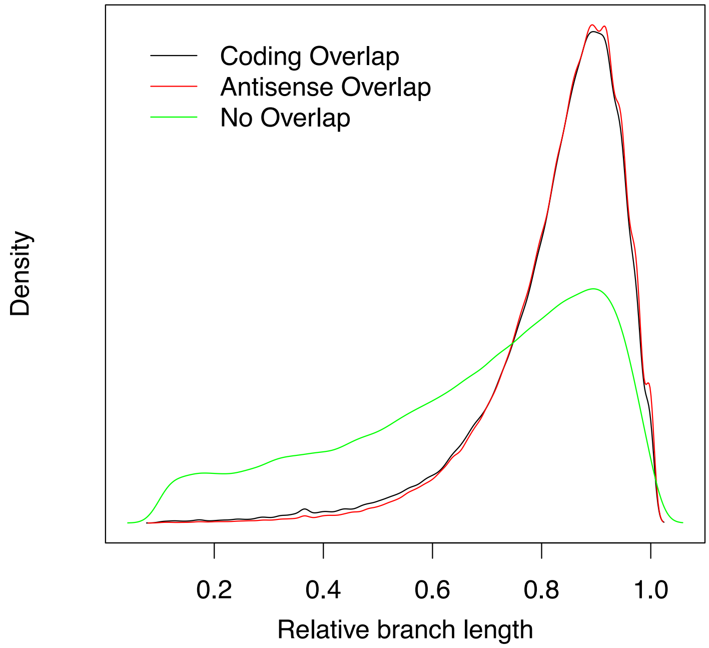

Supplemental Fig. S2. PhyloCSF specifically distinguishes protein-coding conservation

UCSC Genome Browser image illustrates how PhyloCSF differs from measures of nucleotide-level conservation such as phyloP and phastCons. In the particular segment of human Chromosome 1 shown, the signal in the PhyloCSF tracks (light green) is a nearly exact match (green boxes) to the coding exons of the *CEP85* models and the top model of *SH3BGRL3* (thick blue rectangles in the GENCODE track), indicating that PhyloCSF specifically detects selection for protein-coding function. In contrast, the phyloP and phastCons tracks (blue and dark green, respectively) include many signals of nucleotide-level conservation in non-coding regions (red boxes and smaller unlabeled regions) because nucleotides can be conserved for many reasons other than protein-coding function. The bottom model of *SH3BGRL3* was previously annotated according to the criteria of the GENCODE project, which allow for 'putative' alternative translations to be described on alternatively spliced transcripts based on their similarity to the canonical CDS. However, there is no independent evidence that the inferred protein isoform exists, and the stop codon is not conserved beyond gorilla. The lack of PhyloCSF signal at the 3' end (purple box) flags this annotation as suspect.

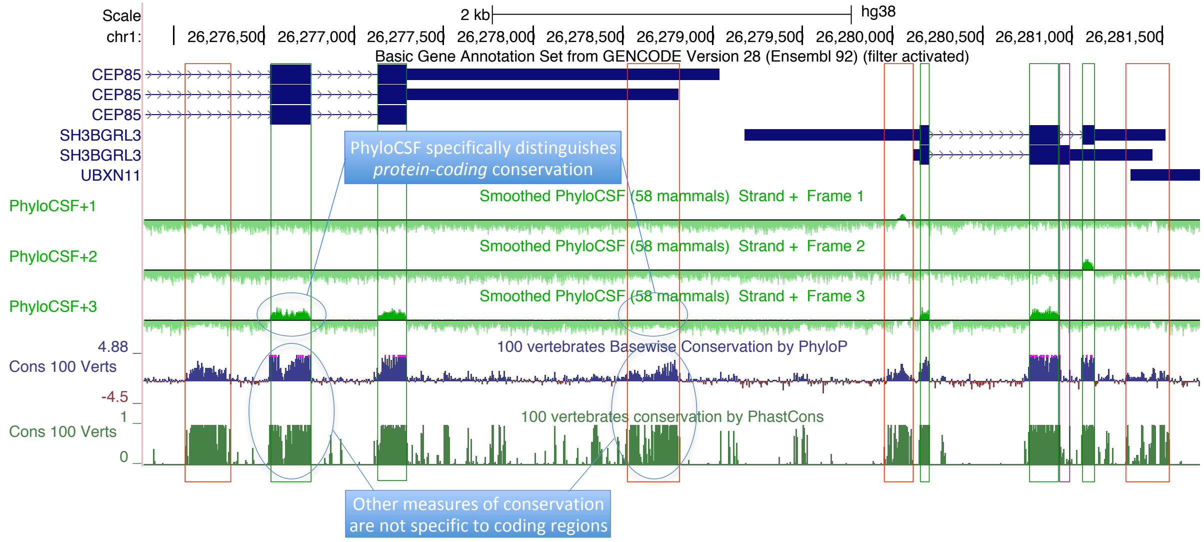

Supplemental Fig. S3. Additional novel genes

Additional novel human loci annotated based on PhyloCSF PCCRs. CDS are shown as open green rectangles, UTRs in red. Additional transcript models have been omitted for clarity. Supporting PCCRs are displayed below the transcript models, with the top rank highlighted. cDNA evidence is shown in brown. RNA-seq-supported introns are shown as dark blue rectangles. Multi-species protein alignments for each locus are collated in Supplemental Fig. S4 (E-I). **(A)** *EDDM13* is a novel human protein-coding gene with a 161-aa CDS identified based on a cluster of PCCRs with a top rank of 609. The gene was previously annotated in GENCODE as an antisense lncRNA on the opposite strand to *ZSCAN5A*. There are no human cDNAs associated with the locus, while EST analysis had led to the annotation of a series of short models without obvious coding potential in GENCODE v22 (two of which have now been re-annotated as nonsense-mediated decay candidate models; purple CDS). The novel CDS is conserved in mammals, and the ENST00000637802 transcript model is supported by a combination of exonic conservation, deep splice site conservation and partial intron coverage in the Intropolis database. Eleven of these exons are 'microexons' under 30 bp in size, which likely explains why just four have associated PCCRs. The locus was previously recognised as protein-coding in mouse (*Epp13*), where full-length cDNA evidence was available. Mouse has an additional 9-bp exon that has apparently lost its transcriptional potential in the human genome due to splice site mutation. FANTOM5 CAGE data indicate that the locus is highly and specifically expressed in epididymis in both human and mouse (data taken from the ZENBU browser, precisely redrawn for clarity), and mouse cDNA AY226991 is derived from epididymis. **(B)** *SMIM41* is a novel human protein-coding gene with a 93-aa CDS, discovered based on a PCCR with a rank of 562. The protein-coding transcript (ENST00000546390) was previously described in GENCODE as a non-coding transcript within the *OR7E47P* pseudogene locus, as the cDNA used to construct the former overlapped with the latter. The novel CDS was found to be conserved within the mammal / avian clade. *SMIM41* and *OR7E47P* are now classified as separate genes in GENCODE. A protein-coding gene was added to mouse GENCODE (*Smim41*) based on cDNA evidence. The untranslated 3' exons of the human and mouse models are non-equivalent, and the mouse genome does not contain an ortholog of the human pseudogene. RNA-seq data from Human Protein Atlas (HPA) and mouse ENCODE indicate that these loci are appreciably expressed in most normal tissues. **(C)** *C1orf232* is a novel human protein-coding gene with a 186-aa CDS, identified based on a PCCR cluster with a top rank of 118. This discovery depended entirely on PhyloCSF, as this region of the genome was previously considered to be untranscribed. Modest short-read support for these introns have now been found in the Intropolis database -- specifically in brain and eye assays -- and the CDS is confidently supported by comparative annotation, with syntenic conservation observed within mammalian and avian genomes. The gene may in fact be older based on homology to fish models that lack synteny (e.g. carp XP_018954235). The novel mouse protein-coding ortholog (*Gm30191*) displays strong FANTOM5 CAGE support specific to inner ear cells (data from the ZENBU browser, precisely redrawn for clarity), and weak RNA-seq support in brain / nervous system experiments in ENCODE datasets. The human FANTOM5 CAGE datasets do not contain any ear-related experiments. **(D)** ENSG00000285043, a novel human protein-coding gene, identified based on a PCCR cluster with a top rank of 226. The 139-aa CDS was identified within the 5' UTR of *ALDOA*. This novel CDS, like *ALDOA*, is apparently conserved amongst vertebrates. In human, it has not been possible to find transcripts covering the novel CDS that do not also utilise exons of the *ALDOA* CDS (e.g. cDNA BC004333), and there is no evidence for polyadenylation to support 3' end annotation prior to the *ALDOA* initiation codon. Although there is no cDNA or long-read evidence to unify the orthologous novel mouse protein-coding gene (ENSMUSG00000114515) with *Aldoa*, short read RNA-seq coverage indicates that transcription routinely continues beyond the currently annotated endpoint of model ENSMUST00000207534 into the *Aldoa* locus. However, *ALDOA* and *Aldoa* share two conserved promoter regions downstream of the novel CDS termination codon (P2 / p2 and P3 / p3), based on FANTOM5 CAGE data in both species; this suggests that, while the novel CDS is not

independently transcribed from *ALDOA* (based on current evidence), *ALDOA* can be transcribed independently of the novel CDS. Zebrafish protein-coding gene ENSDARG00000097765 is clearly a distinct locus to *aldoaa* (though adjacent), including polyadenylation features ('polyA') to confirm its 3' end. FANTOM5 CAGE and RNA-seq data indicate that expression from the promoter of the novel CDS (P1 / p1) is largely limited to testis and cancer cell lines in human and mouse, while *ALDOA* is ubiquitously transcribed at high levels from P2 / p2 and P3 / p3. **(E)** *PFN5P* is a novel unitary pseudogene in human GENCODE, identified based on a PCCR with a rank of 335. It is orthologous to novel mouse protein-coding gene *Pfn5*. Neither the human pseudogene nor the mouse protein-coding gene include any introns; however, their CDS have full-length homology to Profilin 1 and 2 proteins, which are multi-exon loci, indicating that the locus was likely formed by retro-insertion. The human CDS is disabled by a premature termination codon that does not correspond to a known SNP, which is also found in ape genomes. The mouse locus has a testis-specific expression profile (based on ENCODE RNA-seq read coverage), while the human pseudogene is also transcribed in testis (transcript model ENST00000637712 has been annotated based on three ESTs to accompany the pseudogene model ENST00000636088).

S3A. CDS discovered on novel transcript within a previous lncRNA gene

Human *EDDM13:*

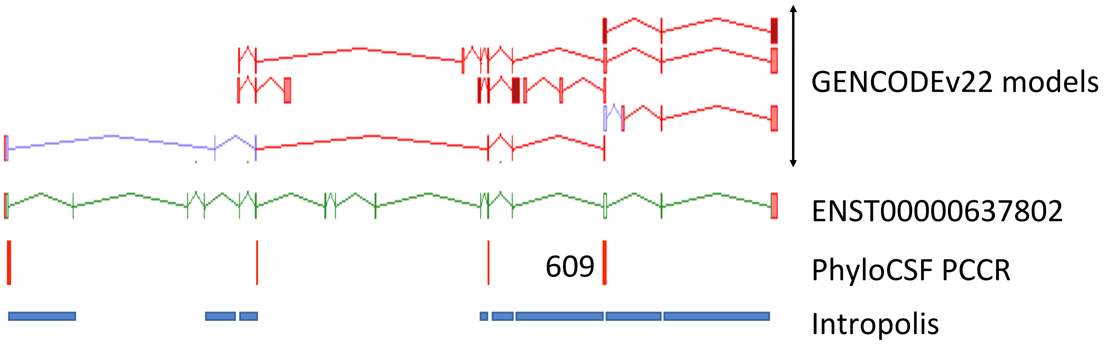

Mouse *Epp13*

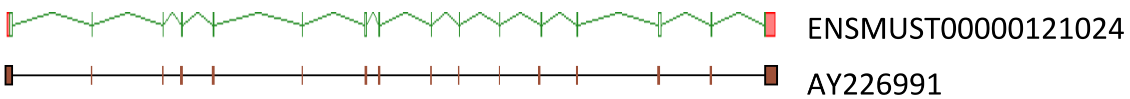

Human CAGE

**Experiment name**	sense strand ->
Epididymis, adult	247.937
Cerebellum, adult, pool1	0.887
Monocyte-derived macrophages	0.813
Testis, adult, pool1	0.758

Mouse CAGE

**Experiment name**	sense strand ->
Epididymis, adult	2076.37
Epidiymis and seminiferous tube, neonate N30	104.004
Universal RNA – normal tissues Biochain	19.525
Clontech Mouse Universal Reference Total RNA	10.0135

S3B. CDS discovered within a pseudogene-overlapping transcript

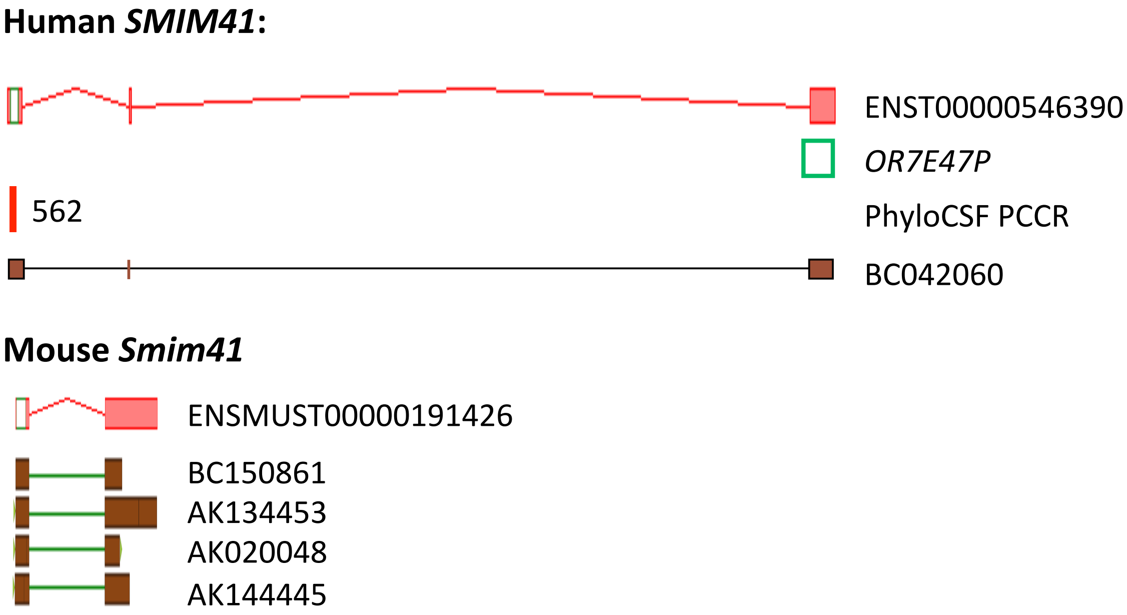

S3C. CDS discovered within a previously unannotated region

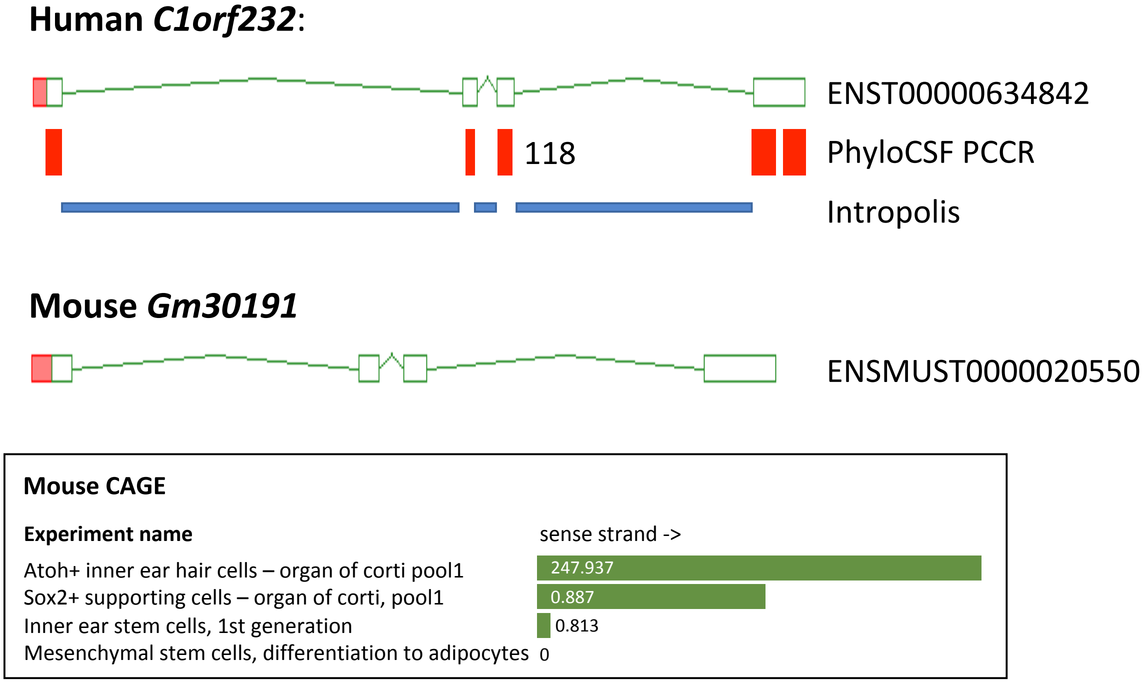

S3D. Novel CDS identified within ALDOA 5’ UTR

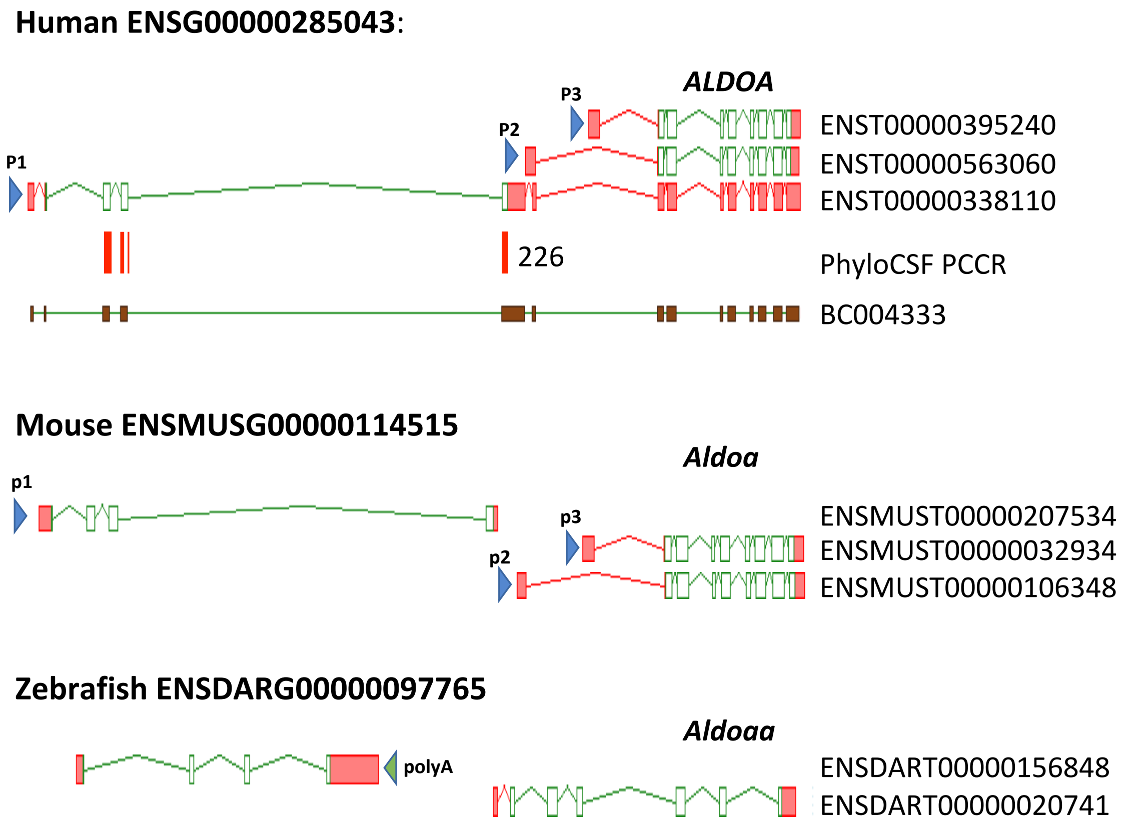

S3E. Novel unitary pseudogene

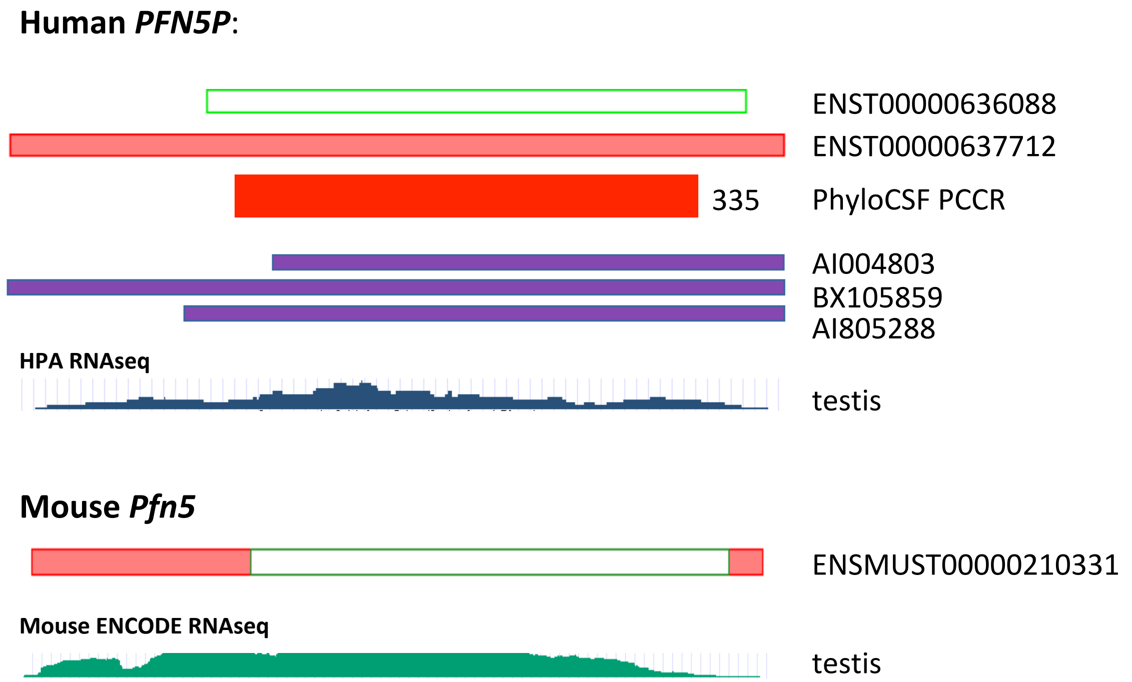

Supplemental Fig. S4. Multi-species protein alignments

Multispecies protein alignments for the gene examples presented in Figure 2 and Supplemental Fig. S3. All alignments were produced using Clustal Omega. **(A)** Alignment for *SMIM31*, Figure 2A. The coelacanth translation ('coe') was taken from PLAR model EnsShortNonCoding|3p|XLOC_155298|TCONS_00200728|0:0.366284|93AA|72AA|(72aa). **(B)** Alignment for *C10orf143*, Figure 2B. The Xenopus translation ('xen') was taken from model OCT69938. **(C)** Alignment for *CCDC201*, Figure 2C. The chicken translation was derived from Ensembl model ENSGALT00000020330. However, manual analysis found that this model incorporates a 5' UTR exon that is poorly supported by transcript data, and removing this exon allowed for the CDS presented here ('chk'). The accuracy of this improved chicken CDS is supported by full-length alignments to unmodified RefSeq models from other bird genomes such as *Numida meleagris* (LOC110390840) and *Meleagris gallopavo* (LOC104910070). The pig translation is from RefSeq model LOC106506856. Although the protein alignment is particularly divergent within the second coding exon, addition confidence in this comparative annotation is provided by the conservation of synteny (anchored by genes *IGFBP1* and *ADCY1*), the conservation of splice sites, and the conservation of reading frame. **(D)** Alignment for *H2BE1*, Figure 2D. The chicken translation ('chk') is taken from existing Ensembl model ENSGAL00000013346. **(E)** Alignment for *EDDM13*, Supplemental Fig. S3A. **(F)** Alignment for *SMIM41*, Supplemental Fig. S3B. The alligator translation ('alg') was derived from model g4669.t1. **(G)** Alignment for *C1orf232*, Supplemental Fig. S3C. The chicken translation ('chk') was taken from RefSeq model XP_429682. **(H)** Alignment for ENSG00000285043, Supplemental Fig. S3D, found within the 5' UTR of *ALDOA*, Supplemental Fig. S3D. The zebrafish translation ('zeb') was taken from Ensembl gene ENSDARG00000097765. **(I)** Alignment for *PFN5P*, Supplemental Fig. S3E. The human locus has been annotated as a unitary pseudogene, with a premature termination codon common to ape genomes indicated by a red asterisk. The dog translation was taken from Ensembl model ENSCAFT00000026220.

S4A. Alignment for *SMIM31*, Figure 2A

```
hum   MELPYTNLEMAFILLAFVIFSLFTLASIYTTPDDSNEEEEHEKKGREKKRKKSEKKKNCSEEEHRI
mus   MELPFTNLEVAFILLAFFIFSLFTLASIYTSPNERNEDDDFHLKEKRRKRKEFKGKKNCSDEEHKI
coe   MALPFTNLEVAFILLAFVVFSIYTLASIYTKPEEKNSEIEEHEREQRMKRRASQIKKQKKMSPDVP
      * **:****:*******.:**::*******.*:: *.: : . : :. **:  : **: . . .

hum   EAVEL-*
mus   ETMQP-*
coe   DANEIN*
      :: :
```

S4B. Alignment for *C10orf143*, Figure 2B

```
hum   MD-------SLALGRWR-QRRAEDLQVPGDVKRVCRRLEASGHE--RGCHQVNACALASW
mus   MD-------SLASGRWR-RRRTEELPAAGDAKRACRRSEPGGYE--CSGHMLTTCALLSW
xen   MDICGDMAMELDLAHVRKRHCMDDIGEFPNSKRTCRGLENLTNGDGILLNQLNECDMVYL
      **       .*  .: * ::  :::    : **.**  *          : :. * :
hum   GPEDRELPSRGCLPAPRPESGQGRLSTGISQNGGRSSAQPCPRCIAGESGHFSHTKNH*
mus   STEDQEPRPR-GLPASQPDCSQERLSSMVLQNGGRSSAQPCLRCISGESGHFNHTDNH*
xen   QMQMA--ANKDSFQTLTPQR-----HQNMVVPGSMGSGQPCPRCIAGESGHINHILGL*
        :      :  : :  *:         :   *. .*.*** ***:*****:.*  .
```

S4C. Alignment for *CCDC201*, Figure 2C

```
hum   MEPG--VQDLGLSSSEDESPSLAIRSPTLRKPLKHSTPEEAALGWSPRPSGGA-------
pig   MELE--AQFPGWSSPEEEALGSGTSQPGPRRLLKHSTPEGAAVSWRSGLLGDVPS----P
chk   MECNTMDSKPDFQMSEEEDSIPNVKRSLKRKLVKHSTPVDSMLSRKTLSLSGPINWLVKD
      **     .  . .  *:*           *: :*****  : :.        ..
hum   ----SYLSGSPMPA-----------------------------HFSQDLASHPAGVSPPA
pig   QE-AGPAGASPLPA-----------------------------PSSQGLPSPRAGLSPTA
chk   QDLSKRAYVSPVPKSTPGRSVAQLSLASVEAYFPYMSPKRFSAVFDLQDSSREISQSSQV
               **:*                                .    *   . *  .
hum   TVRKRRLSTLWASKESSLDLS------APGEEPPTSASLTQRQRQRQQQQQQESLRAKS
pig   PVRKRRLSTIRASGVSSGQLGSDADPWAFEEDPPVPASFTPRRRQRRQQQQD-ASPQARR
chk   VYSGRRLSTVLASDESNEEASEKAVS-SVETQTPTEA-----------SEKIAVTPKSGS
          *****: **  *. : .      :   : *. *           .::   : ::
hum   WAQNPGLPGILNTTGRKRRDPKKRAAAMERVRQWEIYVLQNIEEATQHELTIEDD*
pig   GPPHLGLPGIPNKTSRRRRDLKKLAAALERARQWEIRLLQSIEEATQHELTVQEE*
chk   SWMVSGIPGIKDPPMLKKKKID-KATVRKKQREWVLRQLINIEEATKHELTIEES*
           *:*** :    :::. .  *:. :: *:* :  * .*****:****:::.
```

S4D. Alignment for *H2BE1*, Figure 2D

```
hum   MSAEYGQRQQPGGRGGRSSGNKKSKKRCRRKESYSMYIYKVLKQVHPDIGISAKAMSIMNS
chk   MSAESGRMR----GHPSSSGDKKSKRKPKRKETYSVYIYKVLKQVHPDTGISSKAMSIMNS
      **** *: :        ***:****:: :***:**:************ ***:********
hum   FVNDVFEQLACEAARLAQYSGRTTLTSREVQTAVRLLLPGELAKHAVSEGTKAVTKYTSSK*
chk   FVNDIFERLAVEASRLAQYNHRSTITSREVQTAVRLLLPGELAKHAVSEGTKAVTKYTSSK*
      ****:**:** **:*****. *:*:************************************
```

S4E. Alignment for *EDDM13*, Supplemental Fig. S3A

```
hum   MHRSEPFLKMSLLILLFLGLAEACTPREVATKEKINLLKGIIGLMSRLSPDGLRHNIT
mus   MCRLEPFLKRSLVVLLFLGLAEACVPREVAMEEKIKMLKGILGLMGRLSPDGFRQNII
      * * ***** **::**********.***** :***::****:***.******:*:**
hum   S-LKMPPLVSPQDRTEEE---IKKILGLLSLQVLHEETSGCKEEVKPFSGTTPSRKPL
mus   SSSKTPPLVTTPDKSEEEMKILKRILGLLSLQVLNEETSNCKEEVKPPPATTTVRGL-
      *  * ****:  *::***   :*:**********:****.*******  .**  *
hum   PKRKNTWNFLKCAYMVMTYLFVSYNKGDWCYCHYCNLELDIRDDPCCSF-*
mus   -VRTSGWNFLRCAYMVITFFFVSYNKGDWCYCRYCNPDLDLRDDPCCSFQ*
        *.. ****:*****:*::************:*** :**:********
```

S4F. Alignment for *SMIM41*, Supplemental Fig. S3B

```
hum   MNGSQAGAAAQ-AAWLS-SCCNQSASPPEPPEGPRAVQAVVLGVLSLLVLCGVLFLGGGLLLRAQGL
mus   MNGSQVGAAAK-AAWL--SCCNQSGLPLGPPEGPRMVQAVVLGVLSLLVLCGILFLGGGLLLRAQGL
alg   MNGTVSAEAPTVASLLSTGCAIVVGSEARSQHSVQVVQVAVLCVLCLTVIFGIVFLGCNLLLRAESM
      ***:  . *   *: *  .*.   .      .. : **..** **.* *: *::*** .*****:.:

hum   TALLTREQRASREPEPGSASGEDGDDDS*
mus   IALLARERHSTPEAEPGACGGDDDS---*
alg   MALLARDRRPSKEMEVGIAGT-------*
       ***:*::: : * * * ..
```

S4G. Alignment for *C1orf232*, Supplemental Fig. S3C

```
human  MNQAFWKTYKSKVLQTLSGESEEDLAEERENPA--LVGSETAEPTEETFNPMSQLARRVQ
mouse  MNQAFWKTYKSKVLQTLSGESEENLAEERESPT—-LEESEKAEPTEETFNPMSQLARRVQ
chick  MTQGIWGLYKARVLQTLGGARADGALQEEGEPSELMEAAEPPAVMEEGPGPVSQLARKVQ
       *.*.:*  **::*****.*   :.  :*. .*:  :  :*       **  .*:*****:**
human  GVGVKGWLTMSSLFNKEDEDKLLPSEPCADHPLAARPPSQAAA-AAEARGPGFWDAFASR
mouse  GVGVKGWLTMSSLFNKEDEDKLLPPEPCADHPLAAPPSSQAATAETEPRGPGFWDAFASR
chick  G----GWRTFSSLFTREDEHQLLNAEPCADHPLAAMPAELP----PTQKAGGFWDLFATK
       *    ** *:****.:***.:**  ********** * .      :.    **** **::
human  WQQQQAAAASMLRGTEPTPEPDPEPADEAAEEAAERPESQEAEPVAGFKWGFLTHKLAEM
mouse  WQQQQQAAASMLRGVETAAERDPEPQDKPDEEATECPETREADPAAGFKWGFLTHKLAEM
chick  WQQASAPDKEVAPP-----ELGESPTEPPG---DEGPSDLREPEEGAFRWGFLANKLAEI
       *** .    .:        * . .* :       * *.  .    ..*:****::****:
human  RVKAAPKGD*
mouse  RVKAAPKGD*
chick  RNKNASKGN*
       * * * **:
```

S4H. Alignment for ENSG00000285043, Supplemental Fig. S3D

```
hum  MDASSSPWNPTPAPVSSPPLLLPIPAIVFIAVGIYLLLLGLVLLTRNCLLAQGCCADGSS
zeb  ------------MAVPVWDLPIPLPAIIMISVGLYMVFLAVVLWCHHCLKAK-CDPRCSD
mus  MDASPGPWNPAPASI-GPSLQLPTPAIVFIAVGIYLLLLGLVLLTRHCLLAQGCCTDCSS
                   :    * :* ***::*:**:*:::*.:**  ::** *: *    *.
hum  PCRKQGSSGPPDCCWTCAEACNFPLPSPAHFLDACCPQPTRADWAPRCPRCCPLCDCACT
zeb  CCV---GFSPCEYCLVCAQSCDCRPPSLRSCLDNSCPSPN-----------CTMMDCACT
mus  PCRKQGASETQDCCWSCAEACDFPLPSPTHCLDACCPHLSEAGWAPRCPGCCPLCDCACA
      *    .    : *  **::*:   **    ** .**  .           * : ****:
hum  CQLPDCQSLNCLCFEIKLR*
zeb  CQPPECDSINCICFEIKFK*
mus  CQLPDCQSLNCLCFEIKLR*
     ** *:*:*:**:*****::
```

S4I. Alignment for *PFN5P*, Supplemental Fig. S3E

```
hum  MQCIQDVSEEVWQECLTLFLLTGTCYDAVIITNSPPWLLASYSEGNLFQLTQEEIQILLA
mus  MQSIEDVSHEVWQDCVKMCLQSELCSDAAIITNCPPWLLASFTEGNFTQITQEEIQTLLA
dog  MQCIGDISEEVWQDCITLFLQTGMCCDAAIITNSPPWLLASYPEGNLLQLTQEEIQILLA
     **.* *:*.****:*:.: * :  * **.****.*******: ***: *:****** ***
hum  REGREKLFLQGVTLAGTKCLLI*DNLYTEGNNTMYLRTKGQSQGSRAVTVVQIESVRLVV
mus  REGREKLFLQGITLAGIKCLLIRDNLLTQGN-SMDLRTKGQSRSSQAVTIVQIESVFLVV
dog  REGREKLFLQGITLAGTKCLLIRDNLYTEGNNTMDLRTKGQSRGSQAVTVVQIESVYLVV
     ***********:**** ***** *** *:** :* *******:.*:***:****** ***
hum  IGQKGTEGGPLNLEAFEMAGYVREAIEQHVAHL*
mus  MGKKGTEGGPLNLKAFEVAGYMREAILKKMAHS*
dog  MGQKGTEGGPLNLKAFEMAGYIKEAIHQHMAHF*
     :*:**********:***:***::*** :::**
```

Supplemental Fig. S5. Polymorphism evidence supports recent protein-coding selection.

(A) Fraction of single nucleotide polymorphisms (SNPs) in all newly annotated CDS (left), in the subset consisting of just the 70 novel protein-coding genes (middle), and in a sample of previously annotated CDS (right) that are synonymous when translated in the annotated frame (blue) is larger than the fractions in the theoretical translations in the other two frames (red and green), providing evidence that these CDS, in aggregate, are under purifying selection at the amino acid level in the human population. Error bars show the Standard Error of Mean. Because low-frequency variants have generally been subject to constraint for a shorter time, this analysis was restricted to variants having minor allele frequency greater than 0.01. Of 725 SNPs in new CDS, 281 (39%) would be synonymous if translated in the predicted reading frame, a significant excess compared to the 26% on average if they were translated in one of the other frames (P = 0.0006). When restricted to the 175 SNPS in novel protein-coding genes, 71 (41%) would be synonymous if translated in the predicted reading frame compared to the 27% on average if they were translated in one of the other frames (P = 0.044). By comparison, 51% of SNPs within a sample of previously annotated CDS are synonymous, compared with 21% in alternate reading frames, perhaps indicating a previous bias towards finding and annotating sequences under stronger constraint. **(B)** Cumulative distributions of derived allele frequencies for nonsense (black), missense (red), and synonymous (green) single nucleotide variants (SNVs) in new CDS. Derived allele frequencies for nonsense SNVs are significantly lower than those of missense SNVs (rank sum P = 0.002), which are in turn significantly lower than those of synonymous SNVs (rank sum $P = 3 \times 10^{-8}$). Since purifying selection tends to decrease the frequencies of deleterious derived alleles, this provides further evidence of purifying selection on the amino acid sequences in the human population. Variants were obtained from the Ensembl database (Zerbino et al. 2018). **(C)** Similar cumulative distributions as in (B), but for the subset consisting of just the 70 novel protein-coding genes. The corresponding p-values are 0.14 and 5×10^{-6}, respectively.

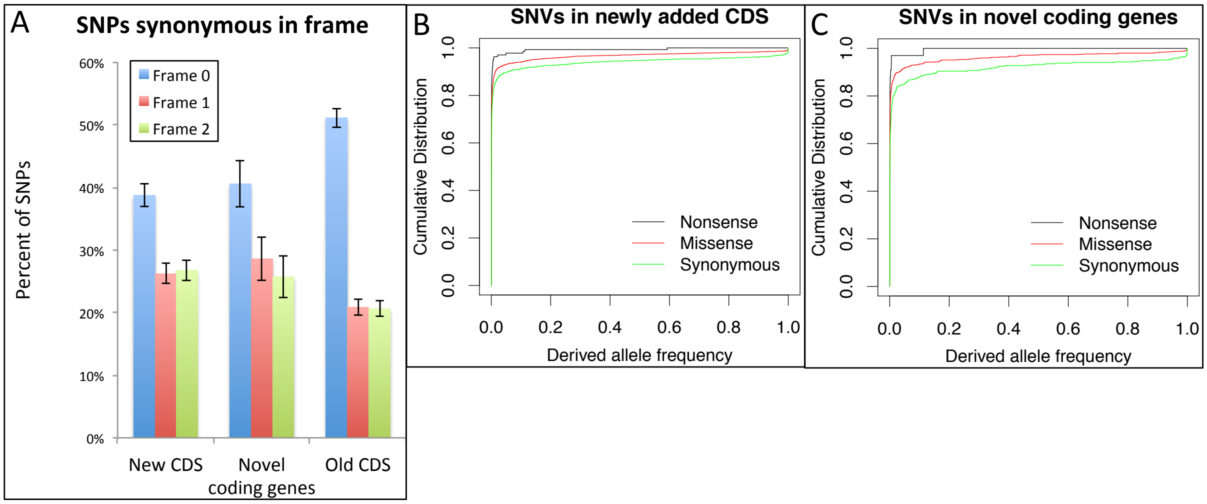

Supplemental Fig. S6. Alignments of the candidate novel CDS in Figure 4

Alignments rendered using CodAlignView. Each alignment includes 10 codons before and after the proposed CDS (gray). Insertions in other species relative to the reference species are hidden for clarity. **(A)** Candidate novel protein coding gene or additional cistron in 5'-UTR of *D. melanogaster nudE* gene. **(B)** 5' extension of an exon of *D. melanogaster* gene *CG33143*, including possible translational readthrough of a TAG stop codon. **(C)** A 1271 amino acid candidate novel protein coding gene in *C. elegans*. **(D)** Three candidate alternative starts for *C. elegans* gene *WBGene00006792* (*unc-58*). In each case, start codon is perfectly conserved, splice site is predicted with high score, and reading frame is preserved. **(E)** Candidate novel first exon of protein coding gene *AGAP005849* in *A. gambiae*. (**F)** Candidate novel protein coding exon in *A. gambiae* gene *AGAP011962*.

Legend

GAC	No Change
GAT	Synonymous
GAA	Conservative
GGG	Radical
TAA	Ochre Stop Codon
TAG	Amber Stop Codon
TGA	Opal Stop Codon
ATG	In-frame ATG
GA-	Indel
GAC	Frame-shifted
<6	Splice Prediction
[]	Exon Break
...	No alignment

S6A. Candidate novel single-exon coding gene in *D. melanogaster*

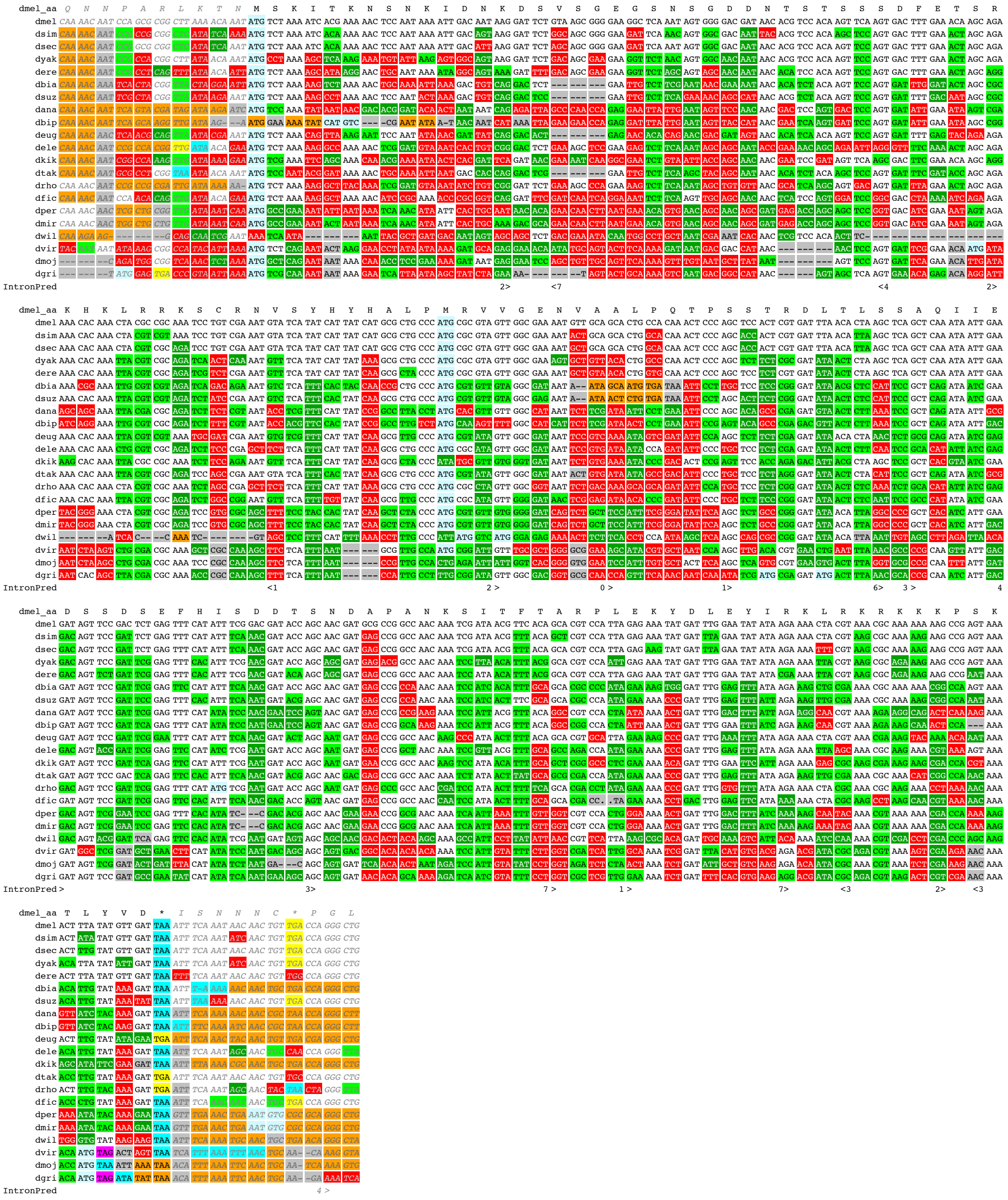

FlyGene_chr3L:9898731-9899168

S6B. Candidate novel exon extension in *D. melanogaster* – possible stop-codon readthrough

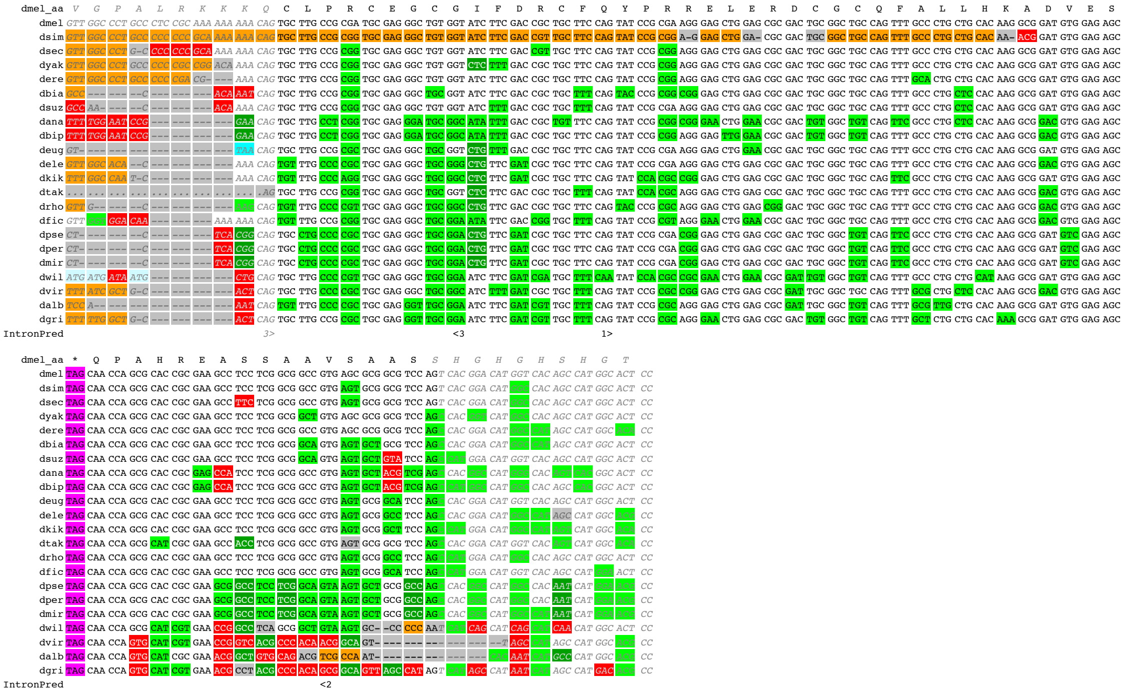

FlyExonExtension_chr2R:22629737-22629909

S6C. Candidate novel 1271-AA coding gene in *C. elegans*

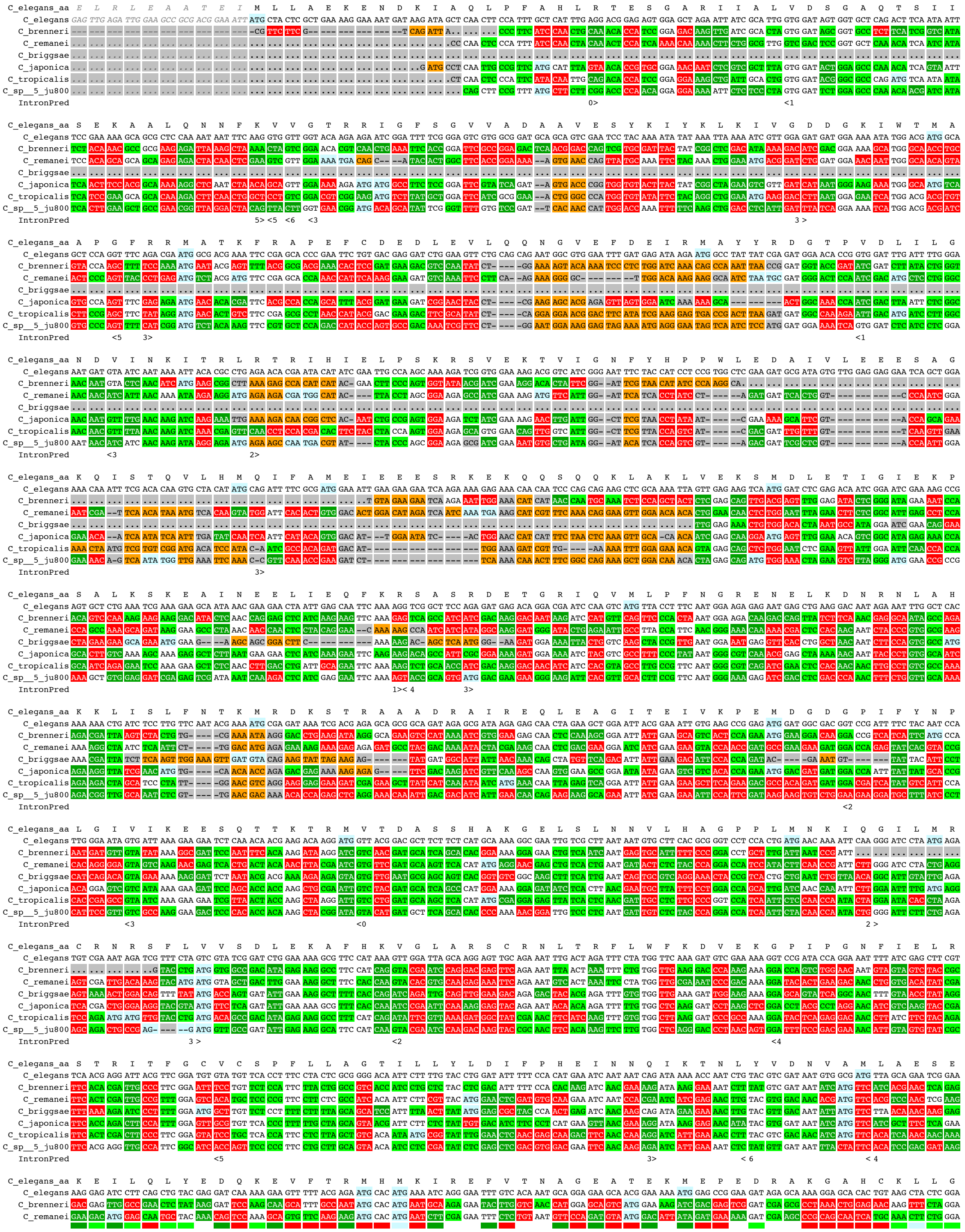

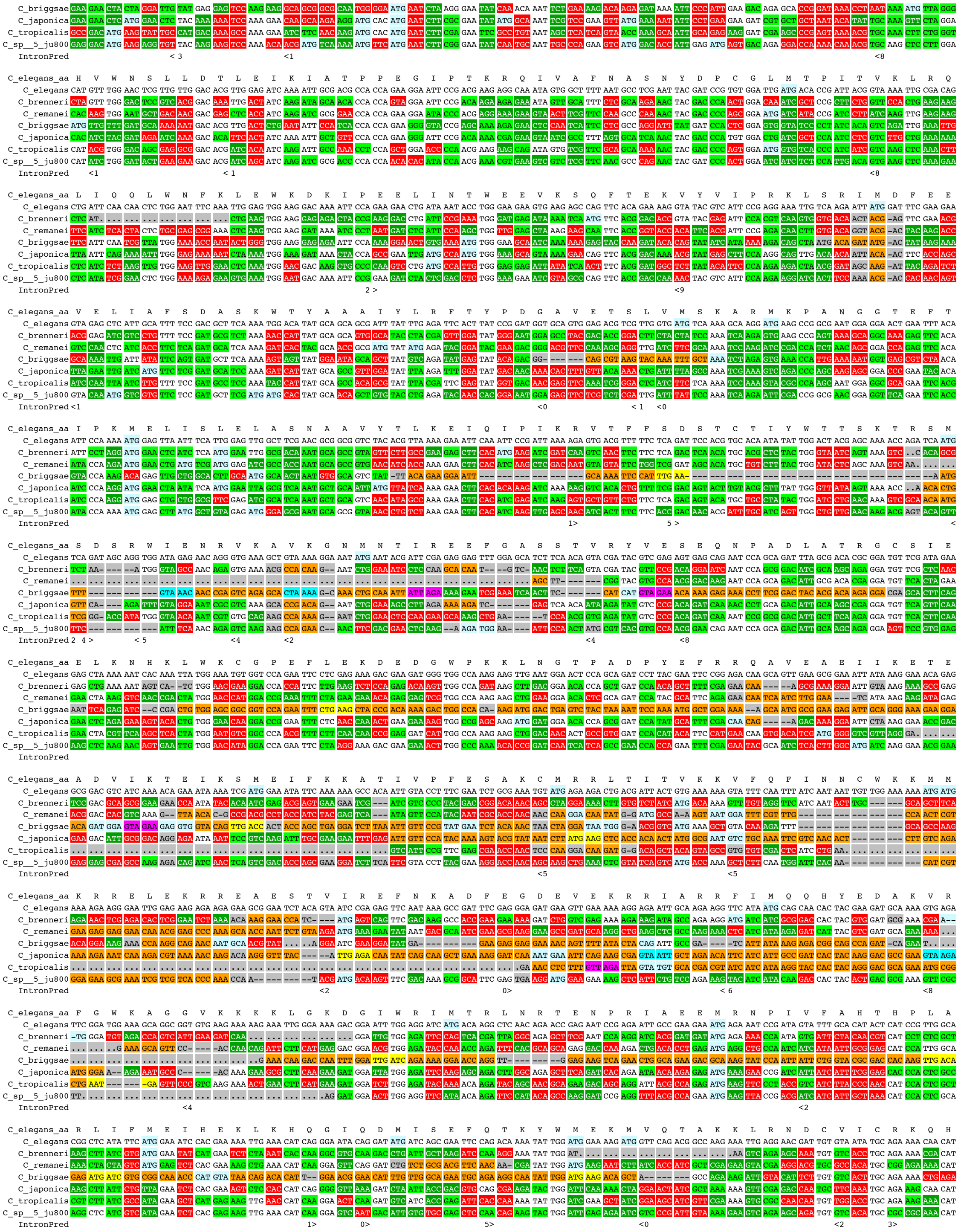

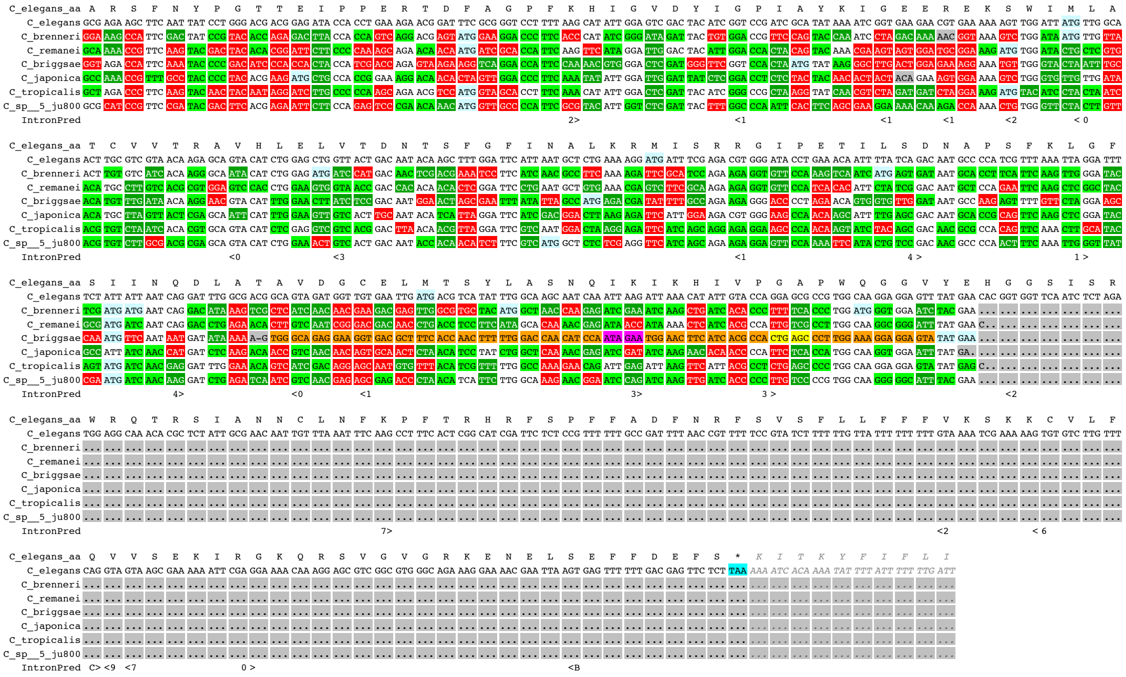

WormGene_chrI:2054512-2058327

S6D. Three candidate novel start exons for *C. elegans* gene *WBGene00006792* (*unc-58*)

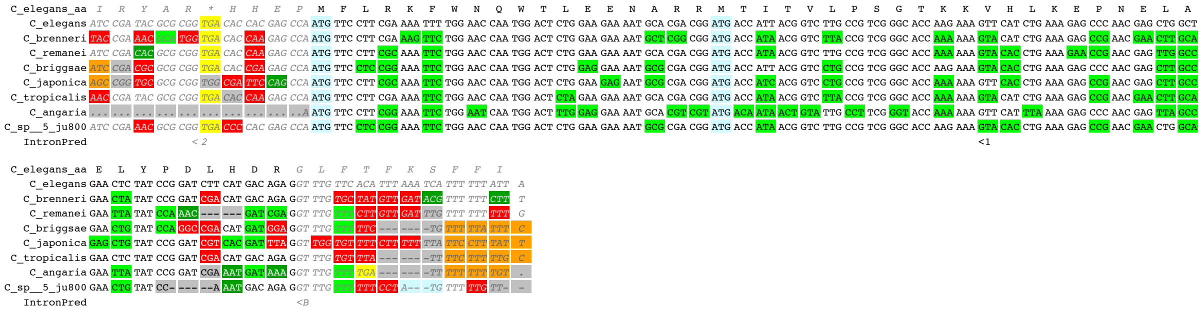

WormExon1_chrX:10117338-10117485

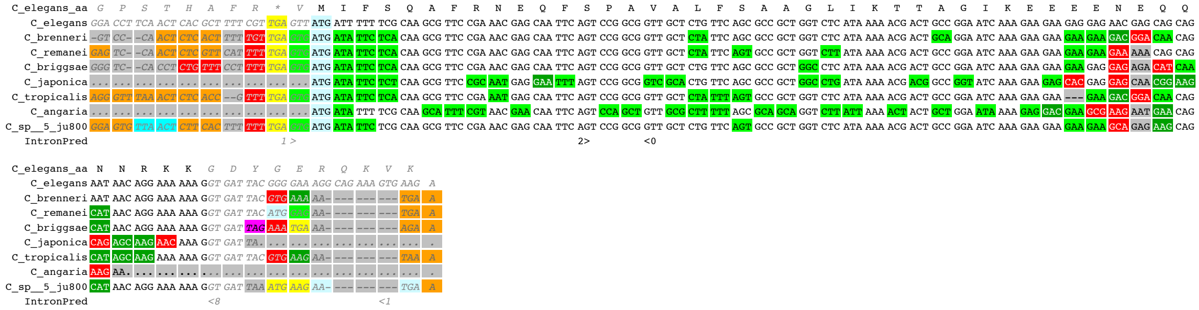

WormExon2_chrX:10113672-10113807

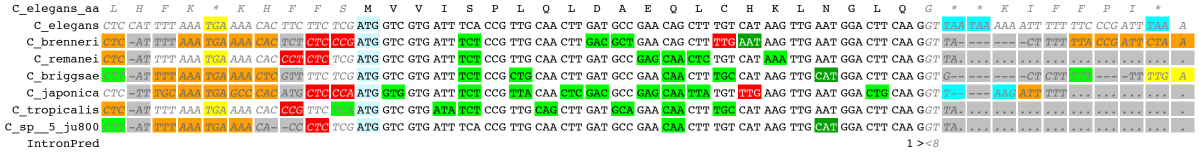

WormExon3_chrX:10111072-10111138

S6E. Candidate novel first exon in *A. gambiae*

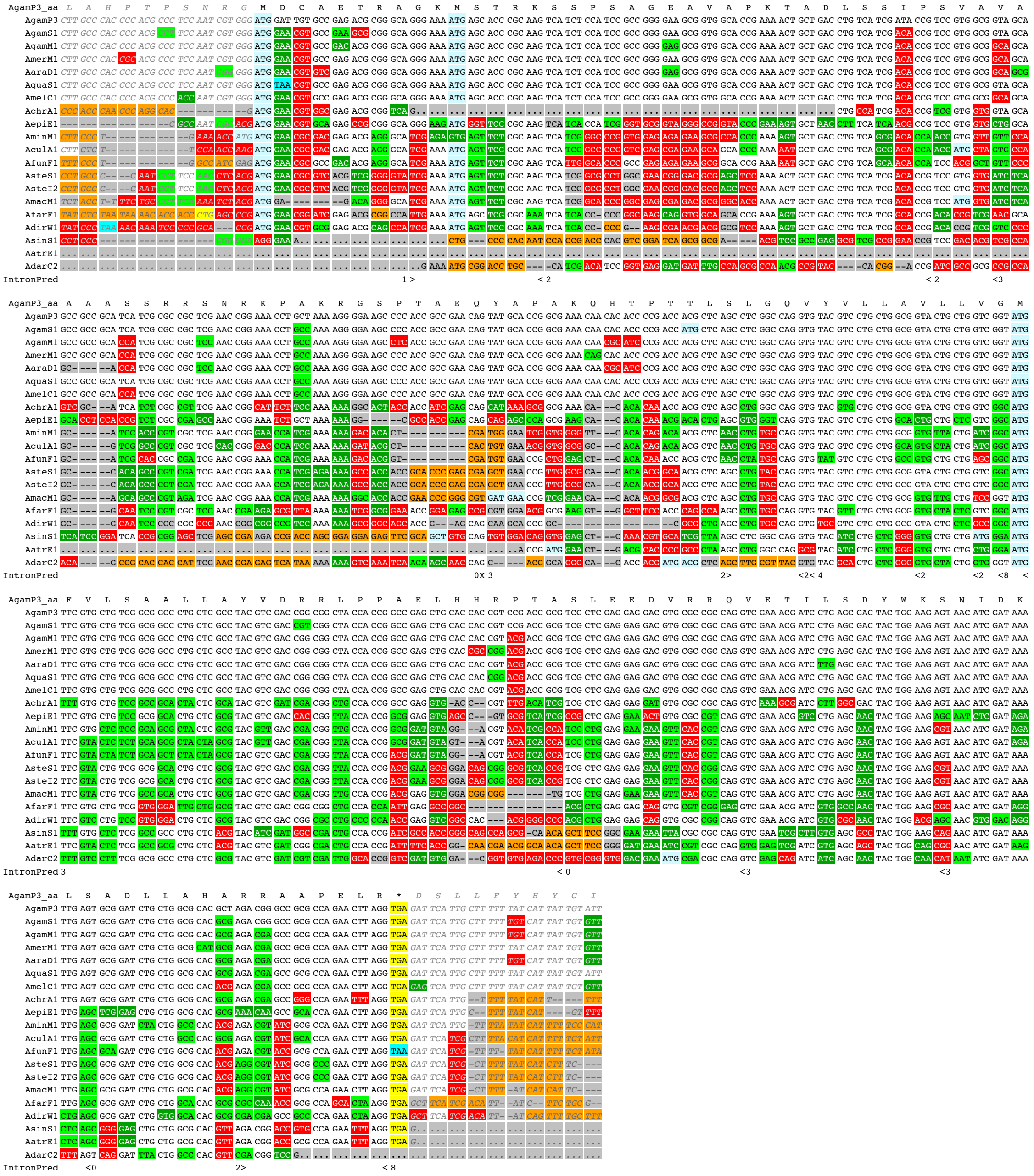

MosquitoGene_chr2L:22539177-22539650

S6F. Candidate novel alternative exon in *A. gambiae*

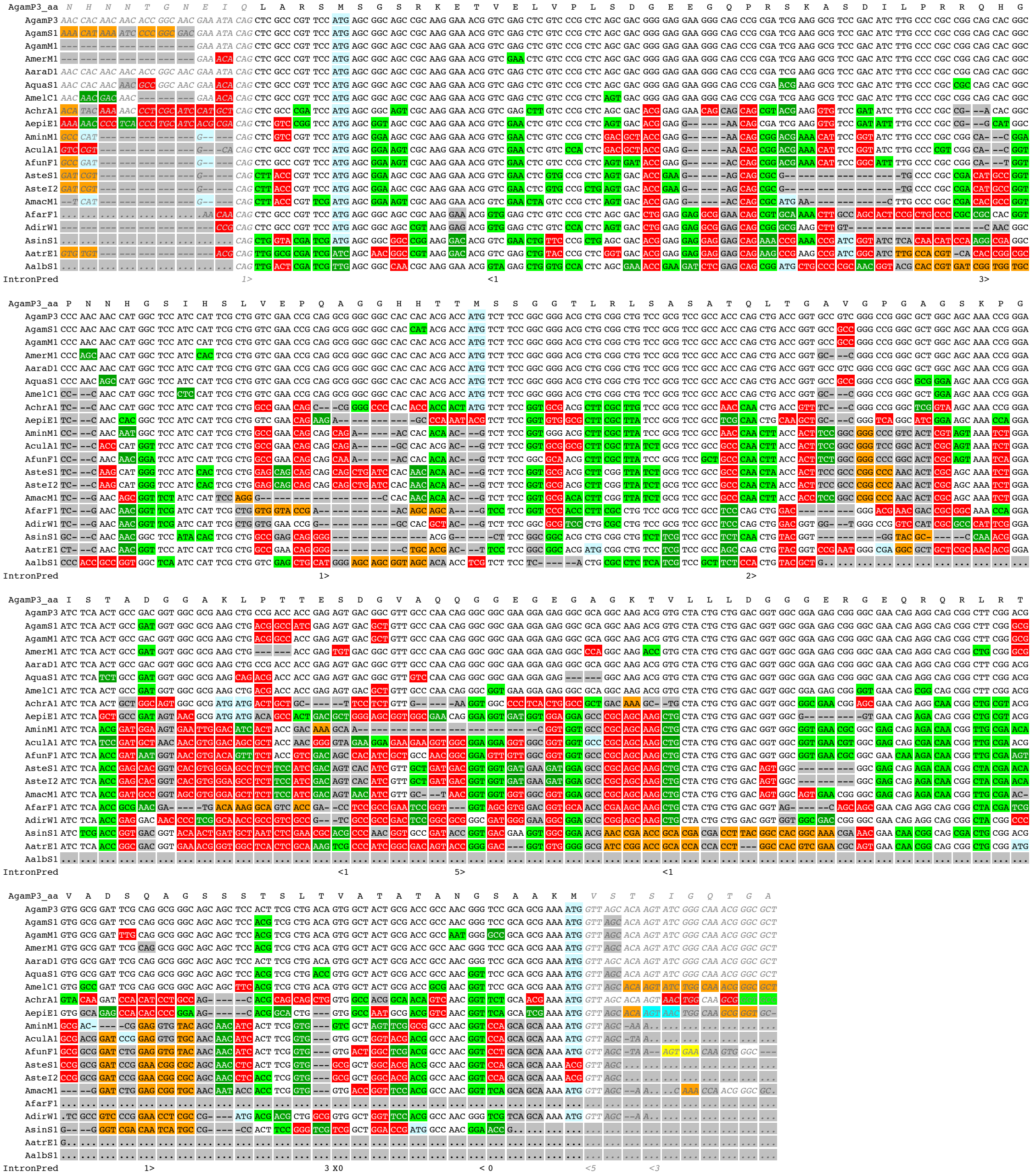

MosquitoExon_chr3L:35635374-35635874

Supplemental Fig. S7. Candidate pseudogenes in *D. melanogaster* and *A. gambiae*

(A) Alignment of *D. melanogaster* candidate novel unitary pseudogene at positions 21837089-21837485 on the '-' strand of Chromosome 2L, identified by a cluster of PCCRs. Propensity of substitutions to be synonymous (light green) and conservative amino acid changes (dark green) shows that this region was protein coding in the *Drosophila* ancestor, but frame shifts (orange) and in-frame stop codons (cyan, magenta, and yellow) have disrupted the reading frame in the five species of the *melanogaster* subgroup (*D. melanogaster, D. simulans*, *D. sechellia*, *D. yakuba*, and *D. erecta*) and independently in *D. elegans*. We have found no homologous region within *D. melanogaster* so this is probably a unitary pseudogene. **(B)** A cluster of *A. gambiae* PCCRs (green rectangles) identify a candidate pseudogene at positions 23749550-23752332 on the '+' strand of Chromosome X, an ortholog of protein coding gene *AGAP007772*. The PhyloCSF signal (green wiggle track) changes frame several times and there are several in-frame stop codons (red rectangles in corresponding lines of base position track), indicating that this is a pseudogene.

S7A. Candidate novel unitary pseudogene in *D. melanogaster*

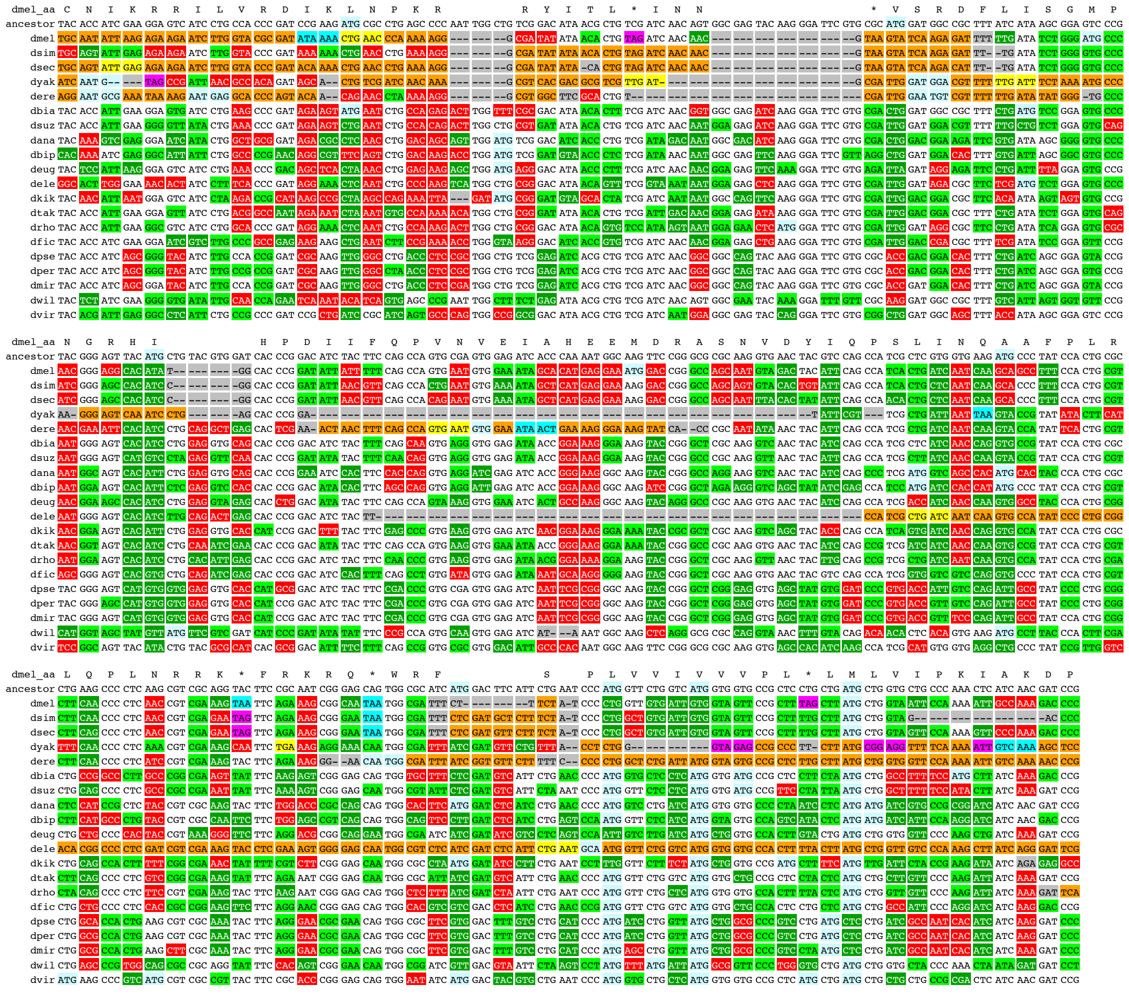

2L:21837089-21837485-

S7B. Candidate pseudogene in *A. gambiae*

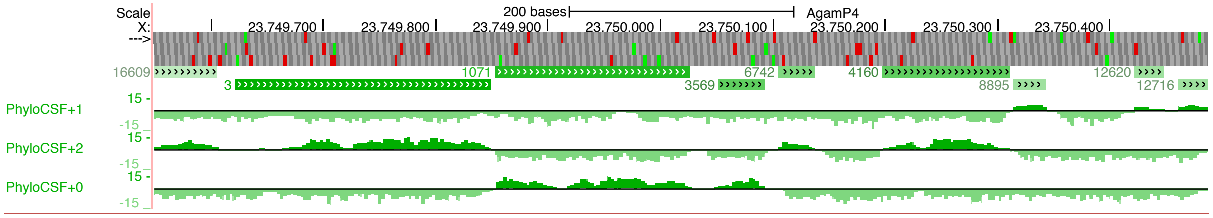

References

Abascal F, Juan D, Jungreis I, Martinez L, Rigau M, Rodriguez JM, Vazquez J, Tress ML. 2018. Loose ends: almost one in five human genes still have unresolved coding status. *Nucleic Acids Res*. http://dx.doi.org/10.1093/nar/gky587.

Adams D, Altucci L, Antonarakis SE, Ballesteros J, Beck S, Bird A, Bock C, Boehm B, Campo E, Caricasole A, et al. 2012. BLUEPRINT to decode the epigenetic signature written in blood. *Nat Biotechnol* **30**: 224–226.

Altschul SF, Gish W, Miller W, Myers EW, Lipman DJ. 1990. Basic local alignment search tool. *J Mol Biol* **215**: 403–410.

Casper J, Zweig AS, Villarreal C, Tyner C, Speir ML, Rosenbloom KR, Raney BJ, Lee CM, Lee BT, Karolchik D, et al. 2017. The UCSC Genome Browser database: 2018 update. *Nucleic Acids Res*. http://dx.doi.org/10.1093/nar/gkx1020.

Derti A, Garrett-Engele P, Macisaac KD, Stevens RC, Sriram S, Chen R, Rohl CA, Johnson JM, Babak T. 2012. A quantitative atlas of polyadenylation in five mammals. *Genome Res* **22**: 1173–1183.

Frankish A, Diekhans M, Ferreira A-M, Johnson R, Jungreis I, Loveland J, Mudge JM, Sisu C, Wright J, Armstrong J, et al. 2018. GENCODE reference annotation for the human and mouse genomes. *Nucleic Acids Res*. https://academic.oup.com/nar/advance-article/doi/10.1093/nar/gky955/5144133.

Giraldo-Calderón GI, Emrich SJ, MacCallum RM, Maslen G, Dialynas E, Topalis P, Ho N, Gesing S, VectorBase Consortium, Madey G, et al. 2015. VectorBase: an updated bioinformatics resource for invertebrate vectors and other organisms related with human diseases. *Nucleic Acids Res* **43**: D707–13.

Gramates LS, Marygold SJ, Santos GD, Urbano J-M, Antonazzo G, Matthews BB, Rey AJ, Tabone CJ, Crosby MA, Emmert DB, et al. 2017. FlyBase at 25: looking to the future. *Nucleic Acids Res* **45**: D663–D671.

GTEx Consortium. 2013. The Genotype-Tissue Expression (GTEx) project. *Nat Genet* **45**: 580–585.

Harrow J, Frankish A, Gonzalez JM, Tapanari E, Diekhans M, Kokocinski F, Aken BL, Barrell D, Zadissa A, Searle S, et al. 2012. GENCODE: the reference human genome annotation for The ENCODE Project. *Genome Res* **22**: 1760–1774.

Hayward A, Ghazal A, Andersson G, Andersson L, Jern P. 2013. ZBED evolution: repeated utilization of DNA transposons as regulators of diverse host functions. *PLoS One* **8**: e59940.

Hezroni H, Koppstein D, Schwartz MG, Avrutin A, Bartel DP, Ulitsky I. 2015. Principles of long noncoding RNA evolution derived from direct comparison of transcriptomes in 17 species. *Cell Rep* **11**: 1110–1122.

Jungreis I, Chan CS, Waterhouse RM, Fields G, Lin MF, Kellis M. 2016. Evolutionary Dynamics of Abundant Stop Codon Readthrough. *Mol Biol Evol* **33**: 3108–3132.

Lagarde J, Uszczynska-Ratajczak B, Carbonell S, Pérez-Lluch S, Abad A, Davis C, Gingeras TR, Frankish A, Harrow J, Guigo R, et al. 2017. High-throughput annotation of full-length long noncoding RNAs with capture long-read sequencing. *Nat Genet* **49**: 1731–1740.

Lee RYN, Howe KL, Harris TW, Arnaboldi V, Cain S, Chan J, Chen WJ, Davis P, Gao S, Grove C, et al. 2018. WormBase 2017: molting into a new stage. *Nucleic Acids Res* **46**: D869–D874.

MacArthur J, Bowler E, Cerezo M, Gil L, Hall P, Hastings E, Junkins H, McMahon A, Milano A, Morales J, et al. 2017. The new NHGRI-EBI Catalog of published genome-wide association studies (GWAS Catalog). *Nucleic Acids Res* **45**: D896–D901.

Mouse ENCODE Consortium, Stamatoyannopoulos JA, Snyder M, Hardison R, Ren B, Gingeras T, Gilbert DM, Groudine M, Bender M, Kaul R, et al. 2012. An encyclopedia of mouse DNA elements (Mouse ENCODE). *Genome Biol* **13**: 418.

Neafsey DE, Waterhouse RM, Abai MR, Aganezov SS, Alekseyev MA, Allen JE, Amon J, Arcà B, Arensburger P, Artemov G, et al. 2015. Highly evolvable malaria vectors: The genomes of 16 Anopheles mosquitoes. *Science* **347**. http://www.sciencemag.org/content/347/6217/1258522.abstract.

Nellore A, Jaffe AE, Fortin J-P, Alquicira-Hernández J, Collado-Torres L, Wang S, Phillips RA III, Karbhari N, Hansen KD, Langmead B, et al. 2016. Human splicing diversity and the extent of unannotated splice junctions across human RNA-seq samples on the Sequence Read Archive. *Genome Biol* **17**: 266.

Potter SC, Luciani A, Eddy SR, Park Y, Lopez R, Finn RD. 2018. HMMER web server: 2018 update. *Nucleic Acids Res* **46**: W200–W204.

Riordan JD, Dupuy AJ. 2013. Domesticated transposable element gene products in human cancer. *Mob Genet Elements* **3**: e26693.

Smit AFA, Hubley R, Green P. 2013. 2013--2015. RepeatMasker Open-4.0.

Sorek R. 2007. The birth of new exons: mechanisms and evolutionary consequences. *RNA* **13**: 1603–1608.

Stanke M, Diekhans M, Baertsch R, Haussler D. 2008. Using native and syntenically mapped cDNA alignments to improve de novo gene finding. *Bioinformatics* **24**: 637–644.

Tedja MS, Wojciechowski R, Hysi PG, Eriksson N, Furlotte NA, Verhoeven VJM, Iglesias AI, Meester-Smoor MA, Tompson SW, Fan Q, et al. 2018. Genome-wide association meta-

analysis highlights light-induced signaling as a driver for refractive error. *Nat Genet* **50**: 834–848.

The FANTOM Consortium and the RIKEN PMI and CLST (DGT), Forrest ARR, Kawaji H, Rehli M, Baillie JK, de Hoon MJL, Haberle V, Lassmann T, Kulakovskiy IV, Lizio M, et al. 2014. A promoter-level mammalian expression atlas. *Nature* **507**: 462–470.

Tilgner H, Jahanbani F, Blauwkamp T, Moshrefi A, Jaeger E, Chen F, Harel I, Bustamante CD, Rasmussen M, Snyder MP. 2015. Comprehensive transcriptome analysis using synthetic long-read sequencing reveals molecular co-association of distant splicing events. *Nat Biotechnol* **33**: 736–742.

Yeo G, Burge CB. 2004. Maximum entropy modeling of short sequence motifs with applications to RNA splicing signals. *J Comput Biol* **11**: 377–394.

Zerbino DR, Achuthan P, Akanni W, Amode MR, Barrell D, Bhai J, Billis K, Cummins C, Gall A, Girón CG, et al. 2018. Ensembl 2018. *Nucleic Acids Res* **46**: D754–D761.
